# Increased Reactive Oxygen Species Formation and Oxidative Stress in Rheumatoid Arthritis

**DOI:** 10.1371/journal.pone.0152925

**Published:** 2016-04-04

**Authors:** Somaiya Mateen, Shagufta Moin, Abdul Qayyum Khan, Atif Zafar, Naureen Fatima

**Affiliations:** 1 Department of Biochemistry, Faculty of Medicine, Jawaharlal Nehru Medical College, Aligarh Muslim University, Aligarh, Uttar Pradesh, India; 2 Department of Orthopedic Surgery, Faculty of Medicine, Jawaharlal Nehru Medical College, Aligarh Muslim University, Aligarh, Uttar Pradesh, India; 3 Department of Biochemistry, Faculty of Life Sciences, Aligarh Muslim University, Aligarh, Uttar Pradesh, India; Alexandria University, EGYPT

## Abstract

**Background:**

Rheumatoid arthritis (RA) is an autoimmune inflammatory disorder. Highly reactive oxygen free radicals are believed to be involved in the pathogenesis of the disease. In this study, RA patients were sub-grouped depending upon the presence or absence of rheumatoid factor, disease activity score and disease duration. RA Patients (120) and healthy controls (53) were evaluated for the oxidant—antioxidant status by monitoring ROS production, biomarkers of lipid peroxidation, protein oxidation and DNA damage. The level of various enzymatic and non-enzymatic antioxidants was also monitored. Correlation analysis was also performed for analysing the association between ROS and various other parameters.

**Methods:**

Intracellular ROS formation, lipid peroxidation (MDA level), protein oxidation (carbonyl level and thiol level) and DNA damage were detected in the blood of RA patients. Antioxidant status was evaluated by FRAP assay, DPPH reduction assay and enzymatic (SOD, catalase, GST, GR) and non-enzymatic (vitamin C and GSH) antioxidants.

**Results:**

RA patients showed a higher ROS production, increased lipid peroxidation, protein oxidation and DNA damage. A significant decline in the ferric reducing ability, DPPH radical quenching ability and the levels of antioxidants has also been observed. Significant correlation has been found between ROS and various other parameters studied.

**Conclusion:**

RA patients showed a marked increase in ROS formation, lipid peroxidation, protein oxidation, DNA damage and decrease in the activity of antioxidant defence system leading to oxidative stress which may contribute to tissue damage and hence to the chronicity of the disease.

## Introduction

Rheumatoid arthritis (RA) is a chronic inflammatory relapsing autoimmune disorder which often affects multiple systems. It is characterised by infiltration of inflammatory cells into the synovium and synovial hyperplasia ultimately leading to the destruction of bone as well as articular cartilage. The prevalance of RA is around 1–2% of the world population with women being affected 3 times more often than men. Although individuals of any age can be affected but the onset is more frequent in 40’s or 50’s. The exact cause of RA remains unkown [1, 2].

A large number of studies have shown that reactive oxygen species (ROS) are implicated in the pathophysiology of many diseases including RA [3, 4]. These are highly reactive chemical species that have the potential to damage lipids, proteins and DNA in joint tissues. Under normal conditions ROS production is controlled by a variety of antioxidant defence system present in the body. The non- enzymatic antioxidant defence includes vitamin A and C, reduced glutathione (GSH) while enzymatic antioxidant includes superoxide dismutase (SOD), catalase, glutathione peroxidase (GPx), glutathione reductase (GR) and glautathione-S-transferase (GST). Imbalance between oxidants and antioxidants due to increased chemical reaction or insufficient antioxidant defence system results in oxidative stress [4]. These ROS if not scavanged properly may damage biological macromolecules [5, 6].

Previous reports suggests the role of oxidative stress in inflammation and destruction in the joints of arthritic animals and RA patients [7]. ROS formation and markers of protein and lipid oxidation has been found to be raised in arthritic animals. The oxidative status has been found to be changed in the serum of RA patients and also in the brain, liver and vascular tissues of rats with experimental arthritis [8].

The present study was designed to determine the significance of oxidative stress and antioxidant status in the blood of RA patients. 2’, 7’-dichlorofluorescein-diacetate (DCFH-DA) assay was performed to monitor intracellular ROS production. To evaluate the extent of oxidative damage on proteins, levels of carbonyl and total sulfhydryl (T-SH) groups were measured. Malondialdehyde (MDA) level was measured as a marker for oxidative lipid damage. Comet assay was done to evaluate the extent of DNA damage. To evaluate the antioxidant status, ferric reducing ability of plasma (FRAP) and DPPH reduction assay were performed. Level of vitamin C, GSH, SOD, catalase, GR and GST were also measured.

Rheumatoid factor which is an antibody against IgG/IgM is found in around 70% of the RA patients. The present study was also aimed to compare the ROS production, concentration of MDA, carbonyl group, DNA damage and antioxidant parameters in seropositive and seronegative RA patients as well as in patients grouped according to their disease activity score (DAS less than or greater than 2.4) and duration of RA—newly diagnosed (symptom duration of less than six months), less than or equal to two years and two to five years.

## Materials and Methods

This work was conducted in the Department of Biochemistry and Department of Orthopaedics, Aligarh Muslim University, Aligarh after the approval from institutional ethical committee of Faculty of Medicine, Jawaharlal Nehru Medical College, A.M.U., Aligarh, 202002. The study was performed in 120 RA patients (37 males and 83 females) who fulfilled the American College of Rheumatology (ACR) / European League Against Rheumatism (EULAR) 2010 classification criteria for RA (Table 1) [9, 10]. Control group consisted of 53 healthy and age matched individuals (17 males and 36 females). Written informed consent was obtained from all the blood donors. The study was conducted for a period of 13 months. Patients with early RA were treated with sulfasalazine (1gm daily), a disease modifying antirheumatic drug (DMARD), deflazacort (6mg daily), a glucocorticoid and aceclofenac (100mg twice daily), a non-steroidal anti-inflammatory drug (NSAID). Patients with more than 2 years of disease were on sulfasalazine (1gm daily) and NSAIDs were given on irregular basis. All the patients and control included in the study were not taking any supplements. Moreover, they were not suffering from any chronic disease nor were smokers nor alcohol consumers.

**Table 1:** Physical characteristics of healthy individual (control) and rheumatoid arthritis (RA) patients included in the study.

	Control	RA patients
**Age**	40.02±11.00	43.11±10.86
**Height (cm)**	160.93±4.57	163.68±7.31
**Weight (pounds)**	130.55±12.98	133.29±13.30
**BMI**	23.22±1.37	23.14±1.66
**ESR (mm/hr)**	13.47±5.45	35.38±10.07*

The values represents the mean ± S.D.

* p < 0.05

DAS which is used for the evaluation of RA activity is calculated by the formula [11]:

DAS=0.53938x√RAI+0.06465x TSJI+0.33x InESR+0.00722x GHA

Total number of tender joints of 53 joints (Ritchie Articular Index [RAI]), swollen joint count of 44 joints (TSJI), erythrocyte sedimentation rate (mm/hour) and general health self-assessment (GHA) by marking a 100-mm visual analog scale (VAS).

### Reagents

2,4,6-Tri-(2-Pyridyl)-5-Triazine (TPTZ), 2, 2-Diphenyl-1-picrylhydrazyl (DPPH), 2′,7′-Dichlorofluorescein diacetate (DCF-DA), Reduced Glutathione, Oxidized Glutathione, Pyragallol, 2,4-Dinitrophenyl Hydrazine (DNPH), RPMI-1640 medium, Trifluoroacetic Acid and Thiobarbituric Acid were obtained from Sigma Aldrich (USA). 5,5’-Dithiobis(2-Nitrobenzoic Acid (DTNB), 1-chloro-2,4-dinitrobenzene (CDNB), ethidium bromide, agarose, Triton X-100 and Nicotinamide Adenine Dinucleotide Phosphate Reduced Tetrasodium Salt (NADPH) were purchased from Sisco Research Laboratories (New Delhi, India). Sulfanilamide and Guanidine Hydrochloride were purchased from Bio Basic Inc. (Ontario, Canada). N-(1-Naphthyl) ethylene diamine dihydrochloride (NED) and Hydrogen Peroxide were obtained from Merck (USA). All other chemicals used in the study were of analytical grade.

### Preparation of blood samples

5 ml of blood was withdrawn from the cubital vein and transferred to vial containing EDTA (an anticoagulant). 2 ml of blood was used for the isolation of lymphocytes by using Histopaque 1077. Cells were suspended in RPMI 1640 and used for detecting DNA damage. Rest of the blood was centrifuged at 2500 rpm for 5 minutes at 4°C. Plasma was removed and stored at -20°C. After removing the buffy coat erythrocytes were washed thrice with phosphate buffered saline (0.01 M phosphate buffer, 0.9% NaCl, pH 7.2). Cells were lysed with 10 volume of ice-cold isotonic saline (pH 7.4) for 2 hours. After centrifugation at 3000 rpm at 4°C hemolysate was obtained. Hemolysates were stored at -20°C until further analysis. Plasma was used for the estimation of vitamin C, FRAP, MDA, NO, protein carbonyl and T-SH. Hemolysate was used for GSH, SOD, catalase, GR and GST estimation.

### Hemoglobin and protein determination

Drabkins reagent from Crest Biosystems (Goa, India) was used to measure hemoglobin present in hemolysate. Protocol of Lowry et al. (1951) was used to calculate protein concentration in plasma with bovine serum albumin as standard [12].

### Dichlorofluorescein assay

DCFH-DA was used to measure the intracellular ROS production in hemolysate by following the method of Keller et al. [13]. The method is based on the formation of highly fluorescent 2’, 7’-dichlorofluorescein (DCF) from nonfluorescent DCFH-DA by ROS. 10 μM of DCFH-DA was added to 5% haematocrit and the reaction mixture was incubated for 1 hour at 37°C. Spectrofluorometer was used to record the fluorescence intensity with an excitation and emission wavelength at 485 and 530 nm respectively.

### Determination of malondialdehyde, carbonyl content and total sulfhydryl groups in plasma

Method of Beuge and Aust [14] was followed to determine the extent of lipid peroxidation (LPO) in terms of malondialdehyde which is an end product of LPO. Plasma was mixed with thiobarbituric acid (0.375% w/v)–trichloroacetic acid (15% w/v)—hydrochloric acid (0.25 N) reagent in the ratio of 1:2. After heating the mixture in boiling water bath for 15 min the precipitate was removed by centrifugation at 1000g for 10 min (after cooling). Absorbance of pink coloured supernatant was recorded at 535 nm. Molar extinction coefficient of 1.56 x 10^5^ M^−1^cm^−1^ was used for the determination of MDA. Results are expressed as nmoles per ml of plasma.

Protocol of Reznick [15] was used for the determination of carbonyl content. Plasma and 10 mM dinitrophenylhydrazine were mixed in the ratio of 2:1 and the mixture was left in dark for 1 hour at room temperature. After adding tricholoroacetic acid mixture was incubated for 10 min in an ice bucket. After centrifugation at 12,000 g for 15 min, ethanol/ethyl acetate (1:1 v/v) was used to wash the pellet. Pellet was dissolved in 6 M guanidine (pH 2.3) and after vortexing and incubating at 37°C for 10 min absorbance was taken at 340 nm. Molar absorption coefficient of 22,000 M^−1^ cm^−1^ was used for the determination of carbonyl content.

T-SH content of plasma was determined by using the method described by Sedlak and Lindsay [16]. Yellow coloured product formed after reaction with DTNB is measured spectrophotometrically at 412 nm. Total sulfhydryl content was expressed as mM.

### Determination of lymphocyte DNA damage

Comet assay was used to determine endogenous lymphocyte DNA damage by the method of Singh et al. [17] with slight modifications. Lymphocytes were mixed low melting point agarose and immediately transferred to fully frosted slide which was precoated with 1% normal melting agarose. For solification of the agarose, slides were covered with a coverslip and kept on ice for 10 minutes. After removing the cover slips slides were immersed in cold lysis solution for 1 hour, followed by unwinding of DNA in alkaline electrophoretic solution. Electrophoresis was performed at 4°C. Slides were stained with ethidium bromide after neutralizing the DNA with ice cold 0.4 M Tris (pH 7.5). Slides were scored with image analysis system attached to fluorescence microscope. Tail length (migration of DNA from nucleus) was used as the parameter to assess DNA damage.

### Assay of antioxidant status

Antioxidant status was evaluated by FRAP and DPPH reduction assays. In FRAP assay (Benzie and Strain, 1996) [18], 1.5 ml of FRAP reagent (10 mM TPTZ, 20 mM FeCl3, 300 mM acetate buffer, pH 3.6) was mixed with 50μl of plasma. After incubating at room temperature for 15 min, absorbance was taken at 593 nm. Aqueous solution of ferrous sulphate was used as standard.

DPPH reduction assay was performed according to the method of Janaszewaska and Bartosz [19]. 20 μl of lysate was mixed with 480 μl of 10 mM sodium phosphate buffer (pH 7.4). Mixture was incubated at 21°C for 30 minutes after the addition of 500 μl of 0.1 mM solution of DPPH in methanol. After centrifugation at 12000g for 10 min, supernatant was read at 517 nm. Test tube containing 500 μl of buffer and 500 μl of DPPH was taken as reference. Reaction mixture with low absorbance was considered to have high free radical scavenging activity.

### Determination of nitric oxide and vitamin C level in plasma

NO was determined spectrophotometrically by the method described by Greiss [20]. Equal volumes of plasma and greiss reagent (0.1& w/v N-(1-naphthyl) ethylenediamine dihydrochloride and 1% sulfanilamide). Absorbance was read at 540 nm against a reagent blank. A calibration curve with known amounts of sodium nitrite was used for the determination of NO level.

Level of vitamin C was estimated by the method described by Aye Kyaw [21]. Equal volumes of plasma and colour reagent consisting of sodium tungstate, disodium hydrogen phosphate and sulphuric acid were mixed and incubated for 30 min at room temperature. After centrifugation at 3000 rpm for 15 min blue coloured supernatant was read at 700 nm. L-ascorbic acid dissolved in oxalic acid solution was used as standard.

### Determination of reduced glutathione, superoxide dismutase, catalase, glutathione reductase and glutathione-S-transferase in hemolysate

GSH in hemolysate was assayed by the method of Jollow [22]. Hemolysate and sulphosalicylic were mixed in equal volumes to precipitate the protein. Mixture was incubated at 4°C for 1 hour followed by centrifugation at 1200g for 15 min. 0.2 ml of supernatant was mixed with 1.1 ml potassium phosphate buffer (0.1 M, pH 7.4) and 0.2 ml DTNB. Yellow colour obtained was read at 412 nm.

Method of Marklund and Marklund [23] was followed to determine the activity of SOD by measuring the inhibition of auto oxidation of pyrogallol. 10 μl of hemolysate was mixed with 2.9 ml of Tris succinate buffer (0.05 M, pH 8.2). 0.1 ml of 8 mM pyrogallol was added to initiate the reaction and the solution was read for 3 min at 412 nm. One unit of SOD was defined as the amount of SOD necessary to produce 50% inhibition of auto-oxidation of pyrogallol. The specific activity was given in units per mg of hemoglobin.

Method of Aebi [24] was used to determine catalase activity. 1.98 ml of potassium phosphate buffer (50 mM, pH 7.0) was added to 20 μl of hemolysate. Reaction was initiated by adding 1 ml of hydrogen peroxide. Change in absorption was recorded for 3 min at 240 nm. One enzyme unit was defined as the amount of catalase required to decompose 1 μM H_2_0_2_ per min at 25°C.

Activity of GST was assayed by the method of Habig [25]. Reaction was initiated by the addition of 1 mM CDNB to the reaction mixture containing 1 mM GSH, potassium phosphate buffer (0.1 M, pH 6.5) and the sample to be tested. Formation of the CDNB–GSH conjugate was observed by an increase in absorbance at 340nm for 3 min. Enzyme activity was reported as units per mg of haemoglobin.

GR activity was measured according to the method of Carlberg and Mannerick [26]. It was based on the formation of NADP^+^ from NADP during reduction of GSSG to GSH. Reaction was initiated by the addition of 10 mM GSSG to the reaction mixture containing EDTA-sodium phosphate buffer (0.1 M, pH 7.6), 2 mM NADPH and sample to be analysed. Change in absorbance was recorded for 3 min at 340nm. Enzyme activity was expressed as units per mg of haemoglobin.

### Statistical Evaluation

Data was expressed as Mean ± S.D. Differences among controls and RA patients were determined by using one-way ANOVA. Correlations between variables were studied by Pearson correlation analysis. P values of less than 0.05 were considered statistically significant.

## Results

**Fig 1:**
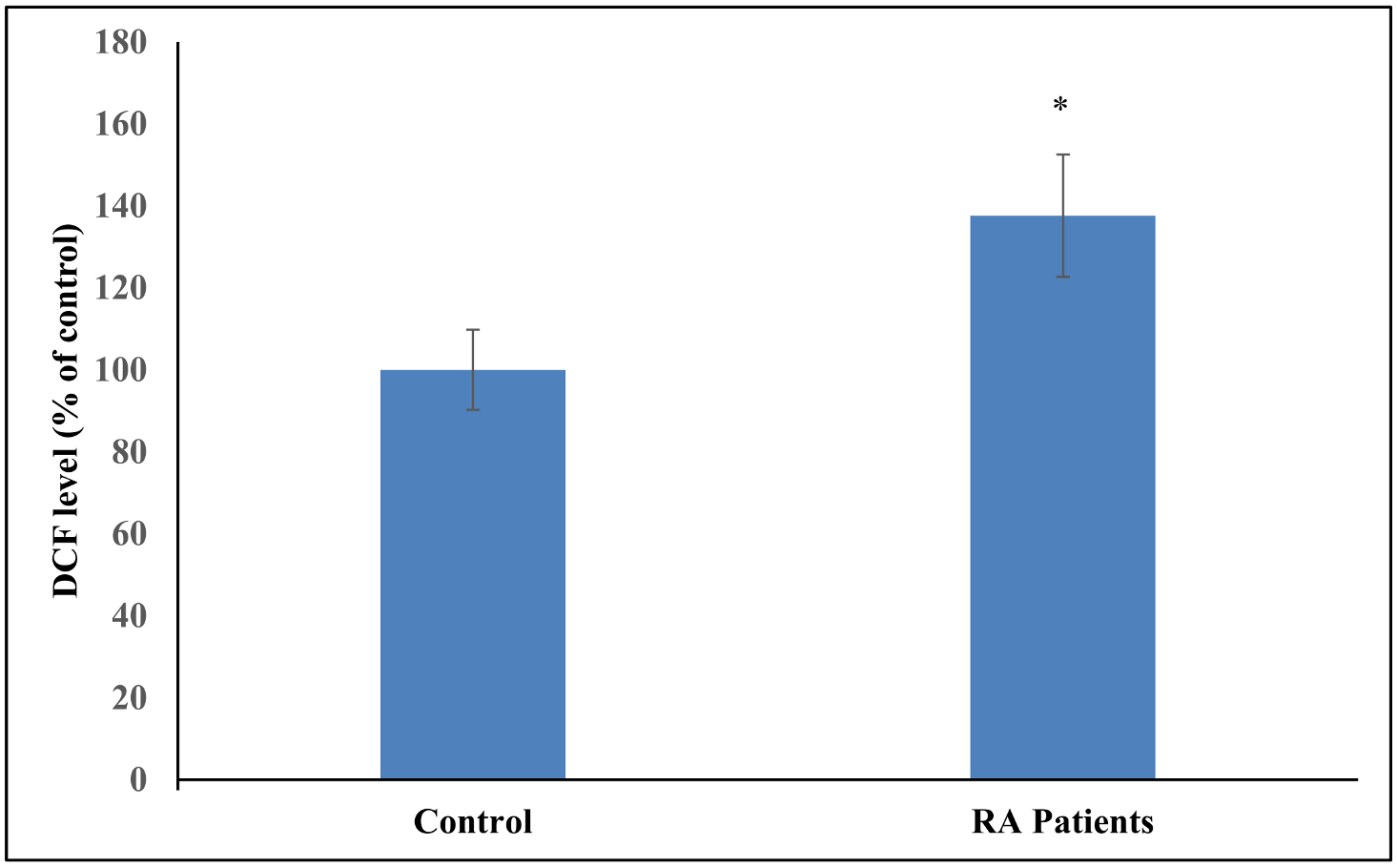
Bar graph representation of data from DCFH-DA assay on lymphocytes of control and RA patients. Fluorescence intensity was recorded at 530 nm using excitation wavelength of 485 nm. Results are mean ± S.D. (*p < 0.05 vs control).

Fig 1 shows the level of DCF in control and RA patients. A significant difference in the level of DCF formed has been observed between the two groups. Table 2 shows the levels of antioxidant stress parameters and the enzymes involved in maintaining antioxidant status in healthy subjects and RA patients. The intracellular antioxidant status of plasma and hemolysate was determined by FRAP and DPPH reduction assays respectively. There occurred a significant decline in the ferric reducing ability and quenching of DPPH radical in plasma and hemolysate (respectively) of RA patients as compared to the healthy individuals. Content of MDA which is a marker of lipid peroxidation was significantly increased in RA individuals than those in controls. Protein oxidation markers (carbonyl and sulfhydryl content) were also significantly different in RA patients as compared to the healthy individuals. Level of nitric oxide in the plasma of RA patients was also found to be significantly increased with respect to control. A significant decrease in the levels of enzymatic antioxidants (SOD, catalase, GR) and non-enzymatic antioxidants (Vitamin C and GSH) were found in RA patients as compared to the control. GST was found to be elevated in RA patients as compared to the healthy controls.

**Table 2:** Malondialdehyde, carbonyl, thiol group, tail length, FRAP, DPPH quenching, NOx, vitamin C, reduced glutathione, superoxide dismutase, catalase, glutathione reductase, glutathione S transferase in control and RA patients.

	Control (n = 53)	RA Patient (n = 120)
**Sex (M/F)**	17/36	37/83
**MDA (nmol/ml)**	1.23±0.24	1.86±0.41*
**Carbonyl (nmoles/mg protein)**	1.07±1.09	1.39±0.14*
**T-SH (μM)**	454.12±54.39	321.39±49.24*
**Tail length (μm)**	4.03±0.63	8.47±1.61*
**FRAP (μM)**	816.64±71.56	619.37±70.12*
**% quenching of DPPH**	57.92±2.25	51.05±3.17*
**NOx (μM)**	8.34±1.68	14.21±0.87*
**Vit C (mg/dl)**	0.99±0.26	0.47±0.17*
**GSH (nmoles/mg Hb)**	5.49±1.23	3.84±1.06*
**SOD (U/mg Hb)**	1.48±0.18	1.05±0.27*
**Catalase (U/mg Hb)**	11.11±1.07	8.77±1.13*
**GR (U/g Hb)**	4.96±0.70	3.13±0.72*
**GST (U/mg Hb)**	0.64±0.69	0.73±0.12*

* p < 0.05

Table 3 shows the correlation coefficient (r) of ROS with various parameters analysed in our study. ROS was found to be significantly and positively correlated with MDA (0.7, p < 0.001), protein carbonyl (0.6, p < 0.001), DNA damage (0.7, p < 0.001), NOx (0.4, p<0.001) and GST (0.3, p < 0.001). Significant negative correlations were found between ROS—T-SH (-0.5, p < 0.001) FRAP (-0.6, p < 0.001), % quenching of DPPH radical (-0.5, p < 0.001), GSH (-0.6, p < 0.001), vitamin C (-0.6, p < 0.001), SOD (-0.7, p < 0.001), catalase (-0.3, p < 0.001) and GR (-0.5, p < 0.001).

**Table 3:** Correlation between the ROS and various biochemical parameters in the RA patients.

Parameters	Correlation coefficient (r)
ROS-MDA	0.7*
ROS-Carbonyl	0.6*
ROS-T-SH	-0.5*
ROS-Tail length	0.7*
ROS-FRAP	-0.6*
ROS-% quenching of DPPH	-0.5*
ROS-NOx	0.4*
ROS-Vit C	-0.6*
ROS-GSH	-0.6*
ROS-SOD	-0.7*
ROS-Catalase	-0.3*
ROS-GR	-0.5*
ROS-GST	0.3*

* p < 0.001

**Fig 2:**
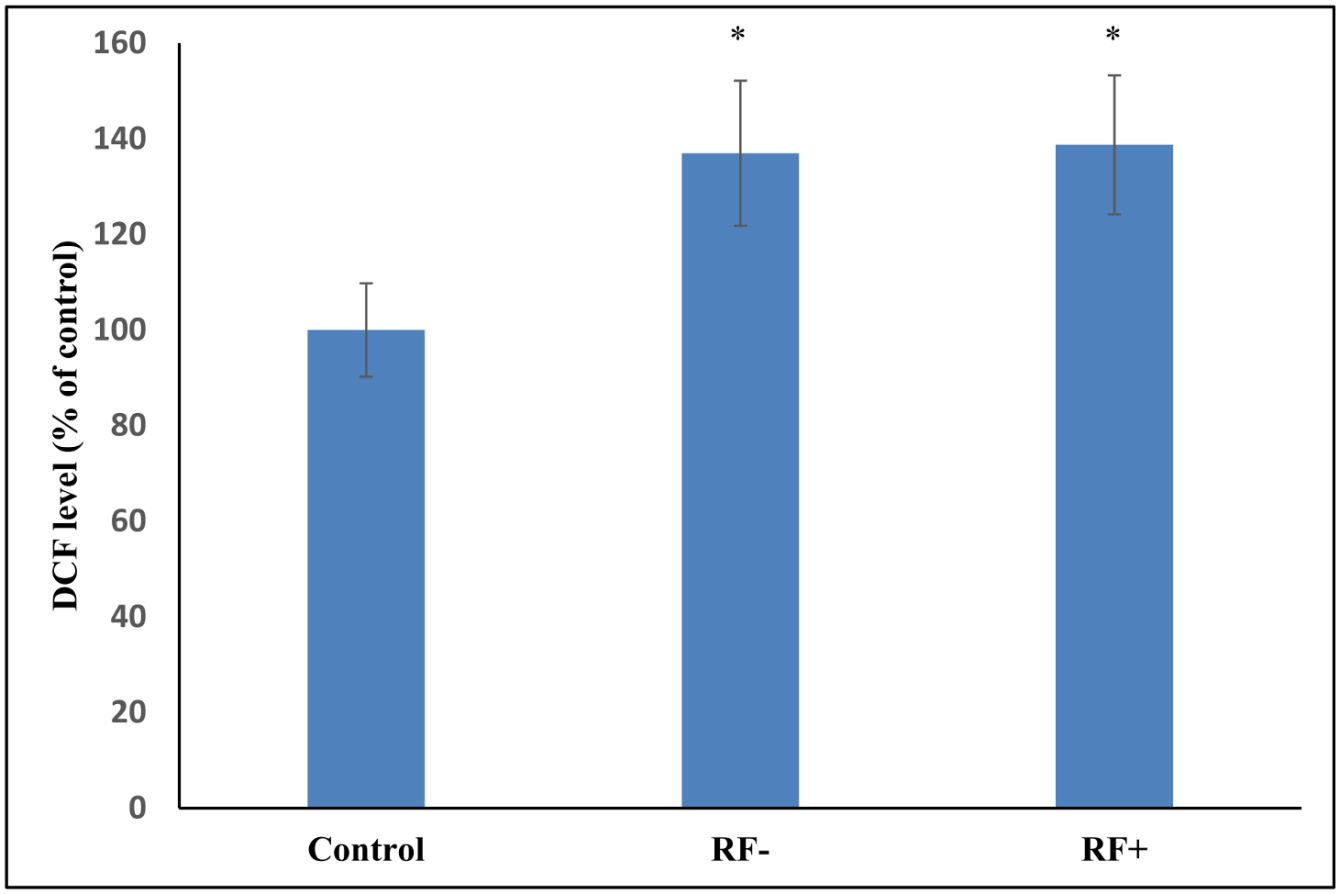
Comparison of the DCF formed in control, RF- and RA+ RA patients. (*p < 0.05 vs control).

Fig 2 shows the level of DCF formed in seropositive and seronegative RA patients. No significant difference has been observed between the two groups. Table 4 shows the levels of antioxidant stress parameters and the enzymes involved in maintaining antioxidant status in seropositive and seronegative RA patients. Patients seropositive for RF had significantly higher MDA content with respect to seronegative RA individuals. A significant reduction in the ferric reducing ability of plasma and quenching of DPPH radical was found in seropositive RA patients as compared to the seronegative RA patients. Tail length was not found to be statistically different in between the two groups. Levels of NOx, Carbonyl, sulfhydryl and enzymatic and non-enzymatic antioxidants (except SOD) were not statistically different in the two groups.

**Table 4:** Comparison of blood plasma and hemolysate parameters in RA patients sub-grouped according to the presence or absence of rheumatoid factor (RF- and RA+).

	RF- (n = 77)	RF+ (n = 43)
**Sex (M/F)**	24/53	13/30
**MDA (nmol/ml)**	1.76±0.39	2.03±0.37*
**Carbonyl (nmoles/mg protein)**	1.38±0.15	1.41±0.11
**T-SH (μM)**	325.94±51.09	313.23±45.17
**Tail length (μm)**	8.29±1.63	8.78±1.55
**FRAP (μM)**	628.96±69.23	602.21±69.20*
**% quenching of DPPH**	51.62±3.13	49.87±2.92*
**NOx (μM)**	14.15±0.92	14.33±0.78
**Vit C (mg/dl)**	0.48±0.17	0.45±0.15
**GSH (nmoles/mg Hb)**	3.91±1.11	3.7±0.95
**SOD (U/mg Hb)**	1.20±0.24	1.09±0.22*
**Catalase (U/mg Hb)**	8.89±0.99	8.56±1.13
**GR (U/g Hb)**	3.19±0.72	3.02±0.71
**GST (U/mg Hb)**	0.72±0.12	0.74±0.11

* p < 0.05

**Fig 3:**
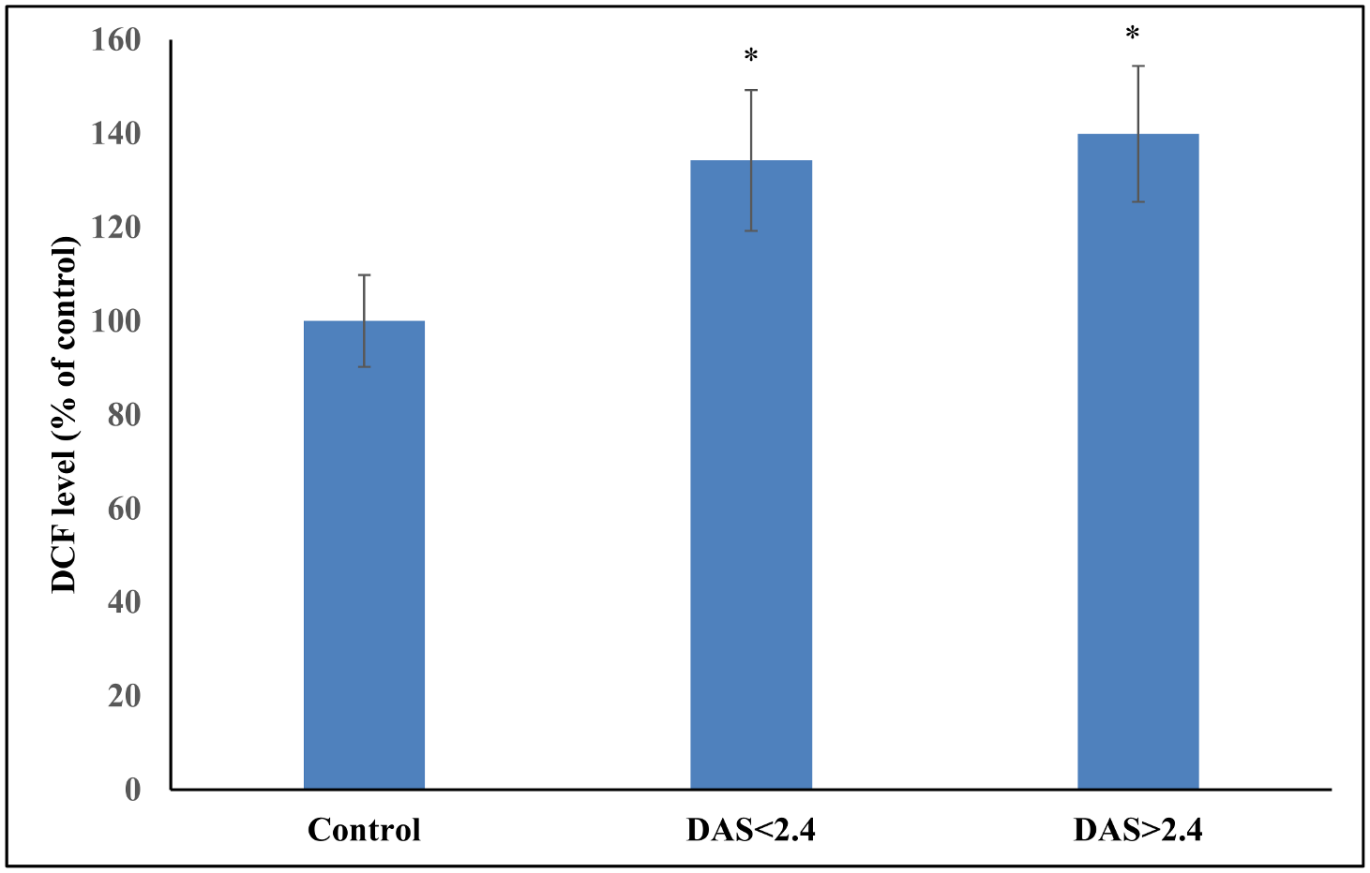
Comparison of the DCF formed in control, RA patients having DAS < 2.4 and DAS > 2.4. (*p < 0.05 vs control).

Table 5 shows the levels of antioxidant stress parameters and the enzymes involved in maintaining antioxidant status in RA patients having disease activity score less than 2.4 and more than 2.4. In RA patients, significantly higher ferric reducing ability and lower DPPH quenching ability was noticed. A marked increase in the ROS production (Fig 3), DNA damage, levels of carbonyl, MDA, NOx and decrease in the sulfhydryl groups were found in active RA patients. There occurred a significant reduction in the level of vitamin C and reduced glutathione in the hemolysate of RA individuals. Also, the levels of SOD, GST, and GR were found to be significantly lower in RA patients having DAS > 2.7 than those RA patients having DAS < 2.7. There was no significant difference between the levels of catalase in RA patients having DAS < / > 2.4.

**Table 5:** Comparison of blood plasma and hemolysate parameters in RA patients sub-grouped according to the disease activity score (DAS).

	DAS<2.4 (n = 49)	DAS>2.4 (n = 71)
**Sex (M/F)**	16/33	21/50
**MDA (nmol/ml)**	1.57±0.31	2.06±0.33*
**Carbonyl (nmoles/mg protein)**	1.36±0.16	1.42±0.11*
**T-SH (μM)**	335.91±48.30	311.36±47.67*
**Tail length (μm)**	7.87±1.6	8.88±1.49*
**FRAP (μM)**	658.86±62.86	592.12±61.68*
**% quenching of DPPH**	53.18±2.71	49.59±2.67*
**NOx (μM)**	13.87±0.88	14.62±0.79*
**Vit C (mg/dl)**	0.54±0.17	0.41±0.14*
**GSH (nmoles/mg Hb)**	4.35±1.05	3.48±0.91*
**SOD (U/mg Hb)**	1.25±0.24	1.09±0.22*
**Catalase (U/mg Hb)**	8.79±1.01	8.76±1.09
**GR (U/g Hb)**	3.39±0.73	2.95±0.66*
**GST (U/mg Hb)**	0.69±0.12	0.76±0.11*

* p < 0.05

**Fig 4:**
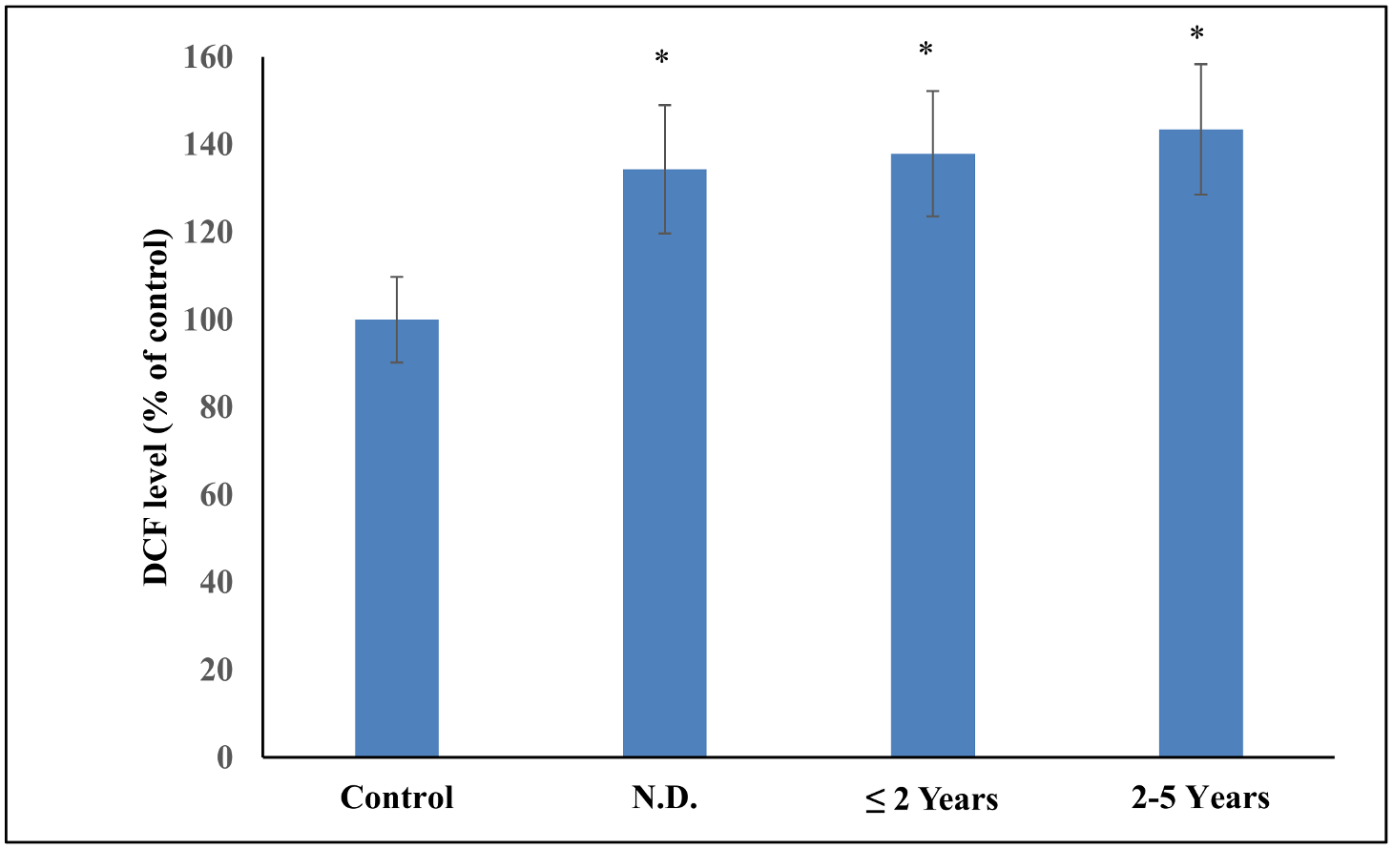
Comparison of the DCF formed in control and RA patients sub-grouped according to the duration of RA: newly diagnosed (ND), less than or equal to 2 years (≤ 2 years) and between 2–5 years. (*p < 0.05 vs control).

Table 6 shows the levels of antioxidant stress parameters and the enzymes involved in maintaining antioxidant status in RA patients sub-grouped according to the duration of RA—newly diagnosed (ND), less than or equal to 2 years and 2–5 years. Comparison of the various blood parameters revealed significantly elevated ROS production (Fig 4), higher DNA damage, elevated carbonyl groups and MDA level and reduction in thiol groups in RA patients sub grouped according to the duration of the disease. There occurred a significant reduction in the ferric reducing ability, DPPH quenching and SOD, GSH and Vitamin C levels in the above mentioned sub groups of RA patients. There was no significant difference in the levels of NOx, catalase and GST in the RA patients sub-grouped according to the disease duration.

**Table 6:** Comparison of blood plasma and hemolysate parameters in RA patients sub-grouped according to the duration of the RA: newly diagnosed (ND), less than years (≤ 2 years) and between 2–5 years.

	ND (n = 53)	≤ 2 Years (n = 39)	2–5 Years (n = 28)
**Sex (M/F)**	18/35	11/28	8/20
**MDA (nmol/ml)**	1.65±0.34	1.93±0.33	2.16±0.40*
**Carbonyl (nmoles/mg protein)**	1.36±0.11	1.39±0.18	1.45±0.11*
**T-SH (μM)**	333.93±47.56	318.43±51.68	301.78±43.05*
**Tail length (μm)**	7.68±1.58	8.60±1.08	9.76±1.41*
**FRAP (μM)**	658.81±64.91	598.81±56.61	573.27±56.42*
**% quenching of DPPH**	53.06±2.62	50.39±2.37	48.17±2.67*
**NOx (μM)**	14.00±0.89	14.36±0.71	14.39±0.98
**Vit C (mg/dl)**	0.51±0.19	0.44±0.13	0.42±0.16*
**GSH (nmoles/mg Hb)**	4.11±1.18	3.68±0.82	3.53±1.00*
**SOD (U/mg Hb)**	1.27±0.24	1.14±0.17	0.96±0.20*
**Catalase (U/mg Hb)**	8.82±1.02	8.81±1.04	8.62±1.15
**GR (U/g Hb)**	3.31±0.83	3.08±0.59	2.87±0.56*
**GST (U/mg Hb)**	0.70±0.13	0.73±0.10	0.77±0.12

* p < 0.05

## Discussion

RA is a relapsing, inflammatory autoimmune disorder with synovial proliferation, destruction of bone and cartilage degradation. Although the etiology of RA is still not very much clear but one recent study clearly demonstrates the involvement of reactive oxygen species in the pathogenesis of the disease. Macrophages and polymorphonuclear cells present at the site of synovitis promotes the formation of reactive oxygen species and subsequent activation of inflammatory molecules which are involved in the progression of RA [4]. Oxidative stress being one of the important cause of various rampant diseases of modern age such as RA, cancer, osteoarthritis, osteoporosis and atherosclerosis, occurs due to the disruption of balance between body’s oxidants load and antioxidants reservoir [27, 28, 29].

The results of our study reveals abundant oxidative stress in RA patients as evidenced by increased intracellular ROS production, increased lipid peroxidation, protein oxidation, DNA damage and impaired enzymatic and non-enzymatic antioxidant defence system of the body. DCFH-DA is a non-fluorescent probe which is commonly used for detecting intracellular ROS production as it can easily pass the cell membrane [13]. DCFH-DA is hydrolysed to DCFH by intracellular esterase which on reacting with ROS forms a highly fluorescent DCF. DCF formation was increased in RA patients relative to control due to enhanced ROS formation thereby indicating insufficient antioxidant defence system in RA patients. ROS formation has also found to be increased in the liver and brain of rats with adjuvant arthritis [30, 31]. Antioxidant status of plasma was determined in terms of ferric reducing antioxidant power. This assay is based on the conversion of ferric to ferrous form by antioxidants present in the plasma of RA patients which serves as reductants. The decrease in ferric reducing ability of plasma of RA patients might be due to the enhanced production of oxidising agents or impairment of the antioxidant defence system of the body. The intracellular antioxidant power of erythrocytes was determined by DPPH reduction assay. Hemolysate of RA patients showed higher quenching of DPPH radical thus indicating compromised state of erythrocytes. Quenching is accompanied with the decolourisation of stable deep purple coloured DPPH free radical in solution.

Nitric oxide is believed to be one of the most important regulator of crucial physiological functions such as immune response, neural communication and blood pressure maintenance [32]. Direct quantification of NO is very difficult as the life is around 0–10 seconds, therefore it is quantified by measuring the levels of stable anions- nitrites and nitrates. A profound increase in the level of NOx in the plasma of RA patients might be due to the hyperactivity of the NO forming enzyme, nitric oxide synthase [33]. The increased NO level is a marker of oxidative stress and similar finding has been reported by other groups [34, 35]. However, Veselinovic et al has even reported unaltered level of NO in the plasma of RA patients [36]. Nitrates and nitrites level have also been reported to be enhanced in the brain and liver of rats with adjuvant arthritis [30, 31].

In the present study, lipid peroxidation was measured in terms of MDA present in blood plasma. The rise in lipid peroxidation product might be due to the increased formation of ROS which tends to increase abundantly during chronic inflammation and hence cause excessive damage to tissues. This is in line with other studies where elevated levels of MDA has been found in the serum, plasma and erythrocytes of RA patients [35, 37, 38, 39]. A significant increase in the lipid peroxidation has also been reported in the liver and brain of rats with adjuvant arthritis [30, 31].

Protein oxidation markers—increased protein carbonyl and decreased sulfhydryl group levels has been found in RA patients. Protein carbonyls are formed either by oxidation of certain amino acid residues or by reaction with lipid peroxidation products. The amount of protein carbonyl groups in plasma proteins reveals the intensity of free radical driven reaction and as well as the extent of protein oxidation. Sulfhydryl groups are responsible for maintaining the structure and function of proteins, enzymes and membranes as well as they can decrease the damage caused by oxidative stress. Our results are in line with other studies where significantly high protein carbonyl and low sulfhydryl group levels has been found in RA patients [5, 40, 41,42] due to the ROS mediated oxidation of proteins. Arthritic rats have also shown increased protein carbonyl groups in the brain and liver [30, 31].

Oxidative stress is also responsible for the oxidation of DNA and increased oxidative DNA damage has been found in many autoimmune disorders [43]. Oxidative stress, in addition impairs DNA mismatch repair system which results in an increase in the formation of DNA adducts in the joints and hence in the augmentation of the disease. [44] Peripheral blood lymphocytes of RA patients showed significantly higher DNA damage as compared to non RA patients.

Neutrophills produce large amount of superoxide anion and hydrogen peroxide which are scavenged by circulating erythrocytes. SOD is regarded as the first line of defence against free radical formation and is required for the dismutation of superoxide radical into hydrogen peroxide and oxygen which otherwise would result in the inactivation of catalase and glutathione peroxidase. The decreased SOD activity might be due to the ROS mediated degradation of SOD during the detoxifying process. However, Jira et al reported that decreased SOD activity may be due to hydrogen peroxide mediated inhibition of the enzyme, which indicates enhanced production of hydrogen peroxide during the dismutation reaction [45]. This decreased erythrocyte SOD activity is in agreement with other studies as well [46, 47]. However increased [36, 48] or even unaltered SOD activity [49] has also been reported by some groups. Catalase catalyses the conversion of hydrogen peroxide into water and oxygen and hence protects the cells from harmful effects of accumulated hydrogen peroxide. Our result that the activity of catalase is lowered in RA patients might be due to the inactivation of catalase by hydrogen peroxide. This result is in line with other findings [48, 46] however some groups have even reported unaltered catalase activity in RA patients [36, 50]. Catalase activity has also been found to be diminished in the brain and liver of arthritic rats [30, 31]. GST is a detoxifying enzyme which conjugates GSH to electrophilic centres present on variety of molecules via –SH group in order to make the latter more water soluble [39]. Our result is in agreement with other studies where raised GST has been reported in the serum and plasma of RA patients [39, 46]. GR catalyses the conversion of GSSG back to GSH by making use of reducing equivalents (NADPH) provided by glucose 6 phosphate dehydrogenase. Riboflavin is of utmost importance for NADP-NADPH cycling but is believed to be deficient in active RA patients and hence the activity of GR is lowered in erythrocytes of diseased individuals [39, 51]. This decreased GR activity is in agreement with our finding.

We observed significantly low levels of non-enzymatic antioxidants (GSH and vitamin C) in RA patients as compared to healthy individuals. GSH, a non-protein sulfhydryl molecule is considered as an important antioxidant defence system in the body. It functions as an intracellular reductant in redox reactions taking place in the human body. It protects cellular components from damage caused by ROS. Low concentration of GSH found in the plasma of RA patients is in agreement with some other studies as well [5, 40, 52]. GSH content has also been reported to be reduced in the liver of arthritic rats [30]. Feri et al [53] reported that leukocyte stimulation oxidises ascorbate more quickly as compared to other antioxidants and hence explains the low level of vitamin C found in RA patients. Low concentration of vitamin C in RA patients has been reported by other groups as well [5, 52].

On evaluating RA patients on the basis of seropositivity and seronegativity of rheumatoid factor, we found a significant difference in the lipid peroxidation, antioxidant status and SOD level in the two groups. Significantly higher serum MDA level in seropositive RA patients has been previously reported by Hassan et al. [39]. On evaluating RA patients on the basis of disease activity, a significant difference in the oxidative stress parameters and the level of antioxidants has been observed. Our results are in agreement with the previous studies where much more compromised antioxidant status of active RA patients has been shown [42, 54]. RA patients sub-grouped according to the disease duration showed a significant difference between the various parameters studied. This suggests that oxidative stress increases with the progression of RA. Another probable reason for this significant difference observed between the three groups is that most of the patients in the third group (2–5 years) have DAS more than 2.4.

## Conclusion

Enhanced ROS production, lipid peroxidation, protein oxidation, DNA damage and failure of antioxidant defence system indicates that there occurs an imbalance between ROS production and elimination leading to the oxidative stress in RA patients which in turn contributes to tissue damage and hence to the chronicity of the disease.

## Supporting Information

S1 File Biochemical parameters of controls and RA patients included in the study. (PDF)
