# Supplementary material for: Increased Reactive Oxygen Species Formation and Oxidative Stress in Rheumatoid Arthritis
Source: PLoS One. 2016 Apr 4;11(4):e0152925. doi: 10.1371/journal.pone.0152925 (PMC4820274; doi:10.1371/journal.pone.0152925)
Supplement: S1 File — (PDF) [file pone.0152925.s001.pdf]

Table A. Biochemical parameters of all the controls included in the study

	MDA (nmol/ml)	Carbonyl (nmoles/mg protein)	T-SH (µM)	Tail length (µm)	FRAP (µM)	% quenching of DPPH	NO (µM)	VITC (mg/dl)	GSH (nmoles/mg Hb)	SOD (U/mg Hb)	Catalase (U/mg Hb)	GR (U/g Hb)	GST (U/mg Hb)
1	1.33	0.98	448.9	3.5	734.67	60.7	12.34	0.8	3.66	1.74	10.04	4.36	0.52
2	1.026	0.97	469	4.2	818.48	61.8	10.35	1.17	6.42	1.56	10.58	5.27	0.43
3	0.962	0.84	527.99	3.6	865.27	59.5	9.56	1.61	6.57	1.28	10.14	4.84	0.4
4	0.98	0.76	420.76	5.4	915.34	60.1	8.64	1.39	4.2	1.38	11.37	4.01	0.61
5	1.192	0.93	482.4	5.2	906.4	59.4	8.23	0.86	5.46	1.85	11.14	5.11	0.54
6	0.916	0.94	360.8	4.2	711.81	58.1	9.45	1.21	5.9	1.43	10.16	6.02	0.56
7	0.987	0.96	417.7	3.6	796.71	56.2	7.76	0.95	4.33	1.65	10.32	5.35	0.59
8	0.98	0.82	534.6	4.5	865.27	55.4	9.23	0.98	7.39	1.36	11.3	4.54	0.42
9	1.288	0.84	427.4	3.7	718.27	55.3	10.29	1.62	6.57	1.59	12.84	4.5	0.65
10	1.096	1.06	415.9	4.1	901.2	56.8	7.43	1.39	6.16	1.84	12.39	4.19	0.67
11	1.038	0.96	368.5	4.9	807.59	59.1	8.16	1.5	5.9	1.27	11.7	5.78	0.42
12	1.243	1.02	512	3.6	743.38	54.1	9.92	0.95	4.78	1.36	10.2	4.03	0.57
13	1.41	0.83	449.7	3.5	716.34	55.9	9.39	1.46	4.26	1.28	10.59	4.12	5.54
14	1.21	0.88	462.8	4.8	974.12	60.5	6.37	0.86	3.09	1.47	10.02	4.56	0.59
15	1.391	0.89	525.8	4.7	824.89	57	7.74	1.02	7.34	1.21	12.94	5.11	0.54
16	1.326	0.85	382.5	3.2	746.86	56.2	5.72	1.15	4.38	1.55	9.84	4.65	0.62
17	1.224	0.93	399.6	4	768.97	58.3	7.48	0.81	4.93	1.37	10.48	5.35	0.66
18	1.5	1.05	455.8	3.5	856.2	55.1	8.59	0.86	6.98	1.59	10.62	5.59	0.56
19	1.467	0.98	536.9	3.7	912.56	56.9	6.39	1.25	5.14	1.57	11.7	6.43	0.54
20	1.051	1.09	432.8	4.5	765.97	58.2	5.27	0.98	6.38	1.42	11.3	4.34	0.53
21	1.108	0.96	476.5	4.1	816.45	62.5	7.43	1.14	7.34	1.57	11.71	4.19	0.59
22	1.083	0.93	375.9	3.8	785.48	56.7	10.49	0.84	4.32	1.59	11.81	5.43	0.6
23	1.339	0.94	512.8	3.4	915.86	59.8	6.94	0.86	4.85	1.38	10.32	6.13	0.57

24	1.704	0.85	527.8	3.2	753.26	54.3	7.36	0.97	7.39	1.49	10.89	4.19	0.53
25	1.019	0.89	487.9	4.1	901.2	55.8	8.21	0.66	6.05	1.27	9.37	4.24	0.58
26	1.23	0.9	468	4.9	821.52	57.9	9.15	0.9	5.8	1.79	12.64	5.23	0.52
27	1.256	0.68	377.4	5.1	843.97	59.8	6.32	1.16	7.39	1.35	12.43	4.16	0.64
28	1.346	0.95	520.8	4.5	715.32	58.3	5.49	0.95	4.69	1.28	11.79	6.23	0.47
29	1.032	1.08	476.9	3.6	843.97	58.4	7.43	0.74	4.28	1.73	12.86	4.53	0.54
30	1.294	0.83	449.5	3.7	768.65	59.6	8.39	0.94	7.28	1.36	11.42	4.56	0.42
31	1.577	0.89	454.9	3.8	821.32	57.5	10.95	0.83	5.17	1.42	9.27	4.34	0.68
32	1.62	0.8	398.9	3.4	939.29	59.7	9.5	1.17	6.87	1.37	10.91	6.02	0.68
33	1.57	0.92	532	4.8	896.34	58.6	6.5	0.81	4.38	1.48	10.63	4.32	0.51
34	1.224	0.74	386.4	4.2	784.23	60.4	8.64	1.16	4.85	1.118	11.42	4.57	0.55
35	0.98	0.91	376.2	3.1	716.24	61.3	7.34	0.96	5.63	1.56	11.3	6.16	0.57
36	1.326	0.97	390.3	3	906.64	60.9	8.33	0.82	3.32	1.5	9.45	4.32	0.62
37	1.75	1.06	498.9	3.8	854.28	58.6	9.34	1.26	7.39	1.37	10.87	4.58	0.52
38	1.544	1.1	412.9	4.7	754.37	59.7	6.95	0.84	5.3	1.63	10.48	5.17	0.46
39	1.62	0.94	475.8	3.1	905.78	58.1	7.49	0.65	5.83	1.43	11.41	4.35	0.47
40	1.59	0.86	487	3.6	865.38	55.7	6.94	1.23	4.16	1.21	11.83	4.87	0.55
41	0.76	0.89	387.7	4.8	765.4	55.7	7.13	1.19	4.24	1.35	9.32	5.67	0.58
42	1.21	0.95	463.2	4.2	876.45	58.4	6.39	0.87	5.36	1.57	11.24	6.21	0.4
43	0.95	0.94	483.23	4.8	724.98	53.7	10.93	0.58	6.58	1.68	12.37	5.35	0.64
44	1.54	1.03	512.38	4.5	798.46	54.6	8.43	0.63	6.34	1.25	9.68	4.32	0.52
45	1.13	0.85	394.24	3.4	912.2	58.2	12.59	0.86	5.13	1.64	12.27	6.21	0.54
46	0.96	8.82	387.63	4.4	734.98	59.6	8.53	1.05	4.54	1.75	12.34	5.37	0.57
47	0.85	0.95	503.42	4.2	856.58	54.9	6.94	1.14	6.78	1.64	9.31	4.83	0.58
48	1.17	1.06	463.87	3.7	843.95	55.3	7.5	1.13	4.08	1.58	9.85	5.17	0.56
49	1.28	0.75	569.54	4.8	725.28	56.5	9.98	0.48	6.76	1.74	12.27	4.76	0.47
50	1.24	0.81	432.12	3.1	749.37	55.5	7.59	0.9	4.34	1.38	13.03	4.98	0.52
51	0.99	0.94	405.83	4.6	765.32	56.7	8.44	0.91	3.54	1.25	10.87	4.34	0.56
52	1.06	1.05	513.85	3.1	834.67	60.4	8.21	0.93	4.35	1.36	11.27	5.67	0.52

53	1.19	0.94	432.84	3.6	734.56	61.9	11.84	0.54	6.57	1.34	12.64	4.21	0.57

Table B. Biochemical parameters of all the RA patients included in the study

	**ROS (% of control)**	**MDA (nmol/ml)**	**Carbonyl (nmoles/mg protein)**	**T-SH (μM)**	**Tail length (μm)**	**FRAP (μM)**	**% quenching of DPPH**	**NO (μM)**	**VITC (mg/dl)**	**GSH (nmoles/mg Hb)**	**SOD (U/mg Hb)**	**Catalase (U/mg Hb)**	**GR (U/g Hb)**	**GST (U/mg Hb)**
1	133.6	2.54	1.54	324.17	7.6	515.9	45.6	12.85	0.31	2.04	0.7	9.14	2.13	0.5
2	168.4	2.76	1.59	248.6	11.6	502.84	45.8	15.57	0.53	4.28	0.72	7.34	2.35	0.85
3	168.3	2.692	1.59	247.9	11.6	506.1	43.5	12.68	0.54	4.38	0.72	9.21	2.09	0.99
4	164.8	2.621	1.53	262.64	11.3	526.79	45.8	15.04	0.57	4.56	0.78	7.94	2.15	0.95
5	164.5	1.993	1.49	302.6	9.5	564.32	48.5	12.85	0.3	2.78	0.94	7.29	2.64	0.72
6	164.3	1.801	1.48	278.72	9.5	614.95	50	14.8	0.26	2.61	0.95	9.87	2.38	0.68
7	163.2	2	1.59	280.4	9.4	557.25	47.28	14.76	0.31	2.84	0.95	8.47	2.43	0.83
8	151.3	1.935	1.49	275.14	9.4	628.56	52.4	14.76	0.5	3.85	0.96	8.48	2.43	0.54
9	162.3	2.224	1.48	278.75	10.3	594.58	51	14.83	0.33	2.97	0.84	7.83	2.43	0.81
10	161.2	2.73	1.55	256.3	9.5	539.3	46.2	15.84	0.21	2.12	0.94	8.74	2.36	0.84
11	159.6	2.32	1.47	286.75	8.4	568.14	46.7	14.83	0.29	2.73	1.2	7.24	0.54	0.76
12	158.9	2.179	1.38	292.12	8.3	543.87	49.6	14.75	0.23	2.34	1.21	7.2	2.35	0.85
13	158.9	2.692	1.5	270.68	12.6	504.54	48.6	15.86	0.24	2.37	0.67	9.64	2.29	0.87
14	158.9	1.391	1.42	276.2	8.6	593.22	53.4	14.75	0.14	4.85	1.16	7.63	3.12	0.52

15	162.9	2.12	1.51	268.31	11.3	632.25	50.6	14.74	0.24	2.38	0.74	8.67	3.13	0.74
16	154.9	2.57	1.41	312.5	8.5	553.99	48.2	14.85	0.21	2.17	1.18	9.28	2.6	0.79
17	154.8	2.179	1. 41	265.46	8.1	650.86	52.6	15.81	0.61	2.79	1.25	7.34	3.17	0.97
18	154.7	2.198	1.64	324.28	11.6	538.24	45.8	15.43	0.28	2.69	0.74	9.28	2.67	0.78
19	154.6	2.17	1.4	315.47	9.5	612.38	49.2	14.95	0.33	2.97	0.94	9.32	2.67	0.77
20	154.3	2.05	1.43	297.48	8.2	648.92	49.8	14.37	0.43	3.46	1.24	9.34	2.96	0.74
21	153.4	1.801	1.52	312.54	10	672.62	48.2	15.64	0.24	2.43	0.87	7.31	3.24	0.72
22	153.1	1.987	1.58	254.15	9.6	510.46	46.2	15.28	0.47	3.68	0.93	8.29	3.06	0.74
23	152.3	1.57	1.42	304.53	7.6	654.32	52.7	14.24	0.26	2.58	1.29	9.53	3.49	0.83
24	152.3	2.57	1.48	278.49	9.5	535.08	50.5	14.87	0.34	3.04	0.94	10.92	3.28	0.69
25	158.7	2.583	1.59	247.32	11.2	513.72	46.9	15.08	0.25	2.46	0.81	7.06	3.34	0.69
26	149.2	2.038	1.49	276.8	10.3	514.81	47.6	15.05	0.4	3.38	0.85	9.72	3.42	0.69
27	148.7	2.57	1.5	269.8	9.6	588.82	49.2	14.85	0.39	3.28	0.94	8.28	3.43	0.69
28	148.7	2.37	1.49	273.7	9.6	537.68	48.9	14.85	0.37	3.18	0.94	8.29	3.43	0.68
29	148.6	1.935	1.46	286.76	8.5	518.08	47.3	14.37	0.41	3.43	1.18	8.29	3.46	0.68
30	148.5	1.28	1.37	329.64	11.8	628.48	54.2	13.62	0.36	3.13	1.3	9.22	4.13	0.82
31	147.8	1.98	1.46	287.14	7.5	612.87	49.8	14.38	0.39	3.38	1.32	7.17	3.47	0.68
32	147.5	2.038	1.37	268	9.8	638.64	50.8	13.67	0.32	2.84	0.9	7.1	3.26	0.72
33	147.5	2.179	1.64	234.1	8.9	581.21	48.4	14.38	0.29	2.74	1.14	7.37	4.03	0.58
34	147.2	2.403	1.52	337.68	10.6	533.32	47.8	14.25	0.21	2.12	0.83	7.63	2.12	0.72

35	146.9	2.435	1.47	284.63	10.6	561.61	46.8	14.21	0.21	2.15	0.83	7.63	2.12	0.97
36	146.9	2.18	1.36	343.34	10.5	631.02	50.2	13.75	0.34	3.08	0.83	7.38	3.65	0.64
37	146.8	1.325	1.37	327.9	9.2	582.39	52.9	13.63	0.34	3.12	1.105	7.39	2.76	0.75
38	145.6	1.62	1.31	359.78	8.3	647.6	55.2	15.62	0.57	4.57	1.23	8.37	4.83	0.79
39	145.2	2.435	1.39	322.8	10.8	568.34	48.6	14.76	0.34	3.12	0.81	9.27	2.67	0.84
40	143.8	2.391	1.63	246.24	9.3	551.82	47.2	13.84	0.45	3.54	1.03	10.82	2.66	0.78
41	143.5	2.403	1.38	324.4	9.6	568.37	46.9	13.84	0.46	3.57	0.91	7.05	2.67	0.78
42	143.5	2.378	1.28	367.8	9.6	535.49	48.7	13.82	0.47	3.62	0.93	11.28	2.67	0.78
43	143.3	1.801	1.48	356.42	9.1	590.76	47.6	13.56	0.48	3.84	1.12	7.18	2.37	0.84
44	142.8	2.378	1.37	334.2	10.7	563.58	48.6	13.28	0.38	3.27	0.82	7.53	4.16	0.54
45	142.6	2.06	1.28	365.64	10.6	607.98	49.5	13.28	0.32	2.94	0.82	7.54	2.67	1.06
46	142.5	1.621	1.32	358.9	9.4	564.32	52.8	14.27	0.52	4.23	0.98	8.42	2.36	0.84
47	142.5	2.05	1.37	332.32	8.3	568.14	48.5	15.7	0.26	2.64	1.22	7.64	3.76	0.64
48	142.5	2.13	1.38	324.53	8.3	574.37	49.8	14.3	0.27	2.64	1.224	7.68	3.78	0.64
49	142.3	1.93	1.57	375.42	10.5	616.03	53.1	14.84	0.28	2.71	0.83	7.41	2.45	0.81
50	142.3	2.15	1.62	265.32	10.5	559.44	45.2	14.84	0.29	2.72	0.84	7.47	2.56	0.79
51	142.3	2.134	1.54	262.64	9.2	555.08	50.5	15.03	0.41	3.42	1.08	7.93	2.19	0.53
52	141.6	2.237	1.42	305.9	9.4	528.92	48.2	14.49	0.23	2.35	0.97	9.62	2.13	0.97
53	138.4	1.929	1.39	321.6	6.5	626.92	52.6	14.46	0.28	2.65	1.39	9.63	2.15	0.95
54	137.5	1.5	1.45	292.12	8.6	635.63	53.5	13.74	0.54	4.52	1.16	7.94	2.15	0.94

55	137.2	1.46	1.39	395.6	7.7	657.89	54.8	13.36	0.47	3.62	1.26	9.38	2.29	0.87
56	136.9	1.891	1.21	304.15	7.7	639.03	52.8	13.29	0.51	3.95	1.26	10.71	2.31	0.87
57	136.8	2.038	1.42	259.96	8.5	537.25	49	13.68	0.53	4.28	1.16	8.12	2.74	0.75
58	136.5	1.891	1.36	343.04	9.2	573.65	49.6	13.73	0.43	3.48	1.1	7.98	2.19	0.87
59	136.4	2.19	1.57	254.6	8.9	543.89	49.6	13.57	0.53	4.32	1.13	10.72	2.37	0.84
60	136.1	2.237	1.34	354.74	8.9	657.94	50.2	13.56	0.43	3.47	1.14	10.73	2.37	0.75
61	135	1.243	1.43	298.7	7.6	703.73	56.2	13.28	0.58	4.73	1.28	10.28	4.73	0.74
62	134.8	2.012	1.58	375.46	10.2	696.58	52.7	14.58	0.52	4.28	0.85	10.14	2.75	0.75
63	134.7	1.76	1.27	385.8	8.2	633.45	52.8	15.27	0.36	3.16	1.24	8.12	2.85	0.75
64	134.5	1.78	1.38	324.56	7.5	543.2	54.1	14.74	0.37	3.16	1.3	10.75	3.29	0.69
65	134.5	1.87	1.35	348.67	8.5	654.13	52.4	14.69	0.34	2.98	1.19	10.81	2.96	0.74
66	134.2	1.737	1.52	267.15	8.8	707.89	52.1	13.52	0.51	3.92	1.15	8.19	2.64	0.79
67	134.2	1.467	1.34	353.95	7.3	719.29	54.9	13.49	0.57	4.64	1.35	8.25	3.12	0.52
68	134.2	2.03	1.35	347.85	9.6	538.98	47.5	15.37	0.54	4.32	1.35	8.11	3.57	0.66
69	134.1	1.544	1.37	332.32	7.4	687.68	54.7	12.79	0.59	4.83	1.34	9.74	4.12	0.57
70	172.5	1.97	1.53	340.36	8.8	682.43	53.4	14.65	0.34	3.08	1.15	8.18	3.14	0.73
71	133.1	1.467	1.37	334.8	8.8	648.96	50.9	14.63	0.53	4.32	1.16	10.52	3.53	0.67
72	132.6	1.243	1.27	376.8	7.9	728.27	52.8	13.27	0.59	4.78	1.26	10.54	4.25	0.54
73	132.5	1.24	1.28	376.5	7.9	602.97	53.7	13.24	0.59	4.8	1.26	8.66	4.32	0.53
74	132.4	1.935	1.32	357.64	7.6	698.76	52.9	15.02	0.25	2.46	1.26	8.26	2.16	0.92

75	132.4	1.621	1.54	257.87	7.6	534.4	49.8	14.98	0.25	2.46	1.26	8.26	2.18	0.89
76	132	1.95	1.28	249.24	9.3	521.92	45.6	14.85	0.55	4.55	1.02	8.37	3.46	0.68
77	131.2	1.942	1.28	368.74	9.3	685.95	52.4	14.85	0.58	4.73	1.02	8.37	3.46	0.68
78	129.8	1.935	1.32	358.6	8.4	785.45	50.3	15.31	0.3	2.76	1.2	8.38	3.47	0.68
79	129.8	1.21	1.28	376.8	7.2	687.98	54.8	14.54	0.64	4.96	1.38	8.14	2.67	0.75
80	129.8	1.692	1.37	328.3	7.6	625.69	50.8	15.27	0.54	4.37	1.27	8.76	3.15	0.73
81	128.6	1.577	1.26	387.4	7.6	581.21	53.1	15.24	0.54	4.4	1.27	8.92	3.16	0.72
82	128.6	1.76	1.26	387.6	7.6	585.56	52.9	15.04	0.64	4.87	1.27	8.93	3.21	0.72
83	128.3	1.628	1.37	332.32	7.4	594.38	53.4	14.35	0.57	4.58	1.34	10.15	3.27	0.7
84	127.8	1.84	1.28	254.7	7.3	584.47	44.2	14.33	0.67	5.14	0.93	10.26	3.28	0.7
85	126.9	1.256	1.28	376.8	7.3	734.67	56.4	13.21	0.73	5.48	1.36	8.14	3.63	0.65
86	126.5	1.692	1.24	392.9	8.2	587.69	51.8	14.37	0.67	5.12	1.24	10.11	2.98	0.74
87	126.5	1.75	1.24	393.69	8.2	632.12	52.5	14.36	0.31	2.84	1.25	10.11	2.98	0.74
88	126.4	1.54	1.51	269.5	8.9	615.7	52.6	12.43	0.64	4.91	1.15	8.56	3.12	0.74
89	126.3	1.487	1.28	367.24	9.9	684.25	52.8	12.38	0.64	4.92	0.87	8.16	2.27	0.74
90	125.9	1.57	1.41	315.2	7.6	637.25	50.7	14.25	0.38	3.27	1.28	9.42	3.25	0.72
91	125.6	1.61	1.39	321	7.6	647.6	54.2	14.25	0.61	4.86	1.29	9.42	3.26	0.72
92	125.6	1.737	1.39	324.28	7.5	713.43	51.6	12.48	0.65	5.12	1.3	9.45	2.95	0.74
93	124.9	1.85	1.14	398.8	7.5	589.45	50.1	14.62	0.45	3.51	1.31	9.48	2.94	0.88
94	124.9	1.23	1.57	254.6	7.2	649.76	52.8	14.84	0.68	5.25	1.38	8.98	3.82	0.64

95	124.8	1.339	1.27	380.98	7.2	684.57	55.5	14.56	0.71	5.27	1.38	9.15	3.85	0.63
96	124.5	1.27	1.44	354.87	9.2	593.18	54.8	14.36	0.71	5.29	1.06	9.16	3.95	0.59
97	124.3	1.628	1.26	387.9	5.9	721.23	56.4	14.36	0.72	5.39	1.5	9.16	3.87	0.62
98	123.5	1.43	1.25	388.6	5.8	756.8	53.4	12.65	0.72	5.42	1.54	9.18	3.94	0.62
99	123.4	1.342	1.25	389.5	5.6	673.25	55.1	12.56	0.72	5.43	1.54	9.21	3.85	0.62
100	123.4	1.54	1.47	286.2	8.5	612.77	55.2	14.28	0.6	4.83	1.18	8.26	2.95	0.74
101	123.3	1.94	1.29	364.2	8.2	594.27	49.5	13.48	0.35	3.12	1.39	9.26	3.48	0.68
102	121.3	1.265	1.19	397.9	6.3	704.56	55.2	12.87	0.67	5.18	1.43	9.37	4.03	0.58
103	120.6	1.27	1.18	398.4	6.2	768.34	55.6	12.85	0.67	5.2	1.45	9.37	4.05	0.57
104	120.5	1.32	1.27	398.6	5.9	674.35	55.6	12.85	0.72	5.45	1.47	8.27	4.12	0.57
105	120	1.54	1.33	248.31	8.2	647.6	53.5	13.54	0.53	4.28	1.24	9.4	4.13	0.54
106	119.5	1.83	1.48	284.08	9.2	612.37	56.5	14.27	0.66	5.12	1.11	8.32	3.49	0.68
107	119.2	1.801	1.37	247.15	6.9	614.95	45.8	15.63	0.41	3.43	1.38	9.12	3.21	0.98
108	116.8	1.358	1.28	368.7	5.3	797.97	56.7	13.46	0.42	3.45	1.64	8.28	3.49	0.67
109	116.2	1.37	1.28	376.43	5.2	699.76	54.7	13.46	0.47	3.83	1.83	8.29	3.52	0.67
110	115.7	1.432	1.41	310.88	6.7	683.45	54.6	12.87	0.48	3.84	1.38	8.93	3.54	0.67
111	115.6	1.467	1.27	376.9	5.6	759.98	58.5	14.83	0.76	5.67	1.57	8.93	3.65	0.64
112	115.4	1.324	1.33	385.48	5.4	698.35	54.2	14.95	0.76	5.71	1.58	8.94	3.67	0.64
113	115.3	1.76	1.23	395.47	5.9	685.67	53.8	13.46	0.78	5.73	1.49	10.38	3.68	0.64
114	115.3	1.57	0.6	410.2	6.4	635.63	50.9	13.75	0.67	5.13	1.43	9.98	3.59	0.65

115	114.6	1.577	1.45	214.4	5.9	695.32	52.1	13.74	0.75	5.64	1.5	10.63	4.37	0.52
116	113.6	1.76	1.27	386.5	6.6	638.33	46.9	13.58	0.52	4.16	1.24	8.29	3.31	0.69
117	113.5	1.75	1.36	342.12	6.6	714.28	52.1	13.57	0.38	3.24	1.39	8.32	4.33	0.53
118	113.5	1.64	1.46	290.5	6.8	615.75	48.9	12.76	0.64	4.87	1.38	8.45	3.64	0.65
119	113.2	1.23	1.17	296.5	5.3	788.9	52.7	13.74	0.81	5.82	1.61	7.47	4.37	0.87
120	113.2	1.62	1.37	340.36	6.5	643.28	52.6	13.23	0.74	5.62	1.41	9.03	4.36	0.74

Table C. Biochemical parameters of seronegative RA patients

	ROS (% of control)	MDA (nmol /ml)	Carbonyl (nmoles/mg protein)	T-SH (µM)	Tail length (µm)	FRAP (µM)	% quenching of DPPH	NO (µM)	VITC (mg/dl)	GSH (nmoles/mg Hb)	SOD (U/mg Hb)	Catalase (U/mg Hb)	GR (U/g Hb)	GST (U/mg Hb)
1	133.6	2.54	1.54	324.17	7.6	515.9	45.6	12.85	0.31	2.04	0.7	9.14	2.13	0.5
2	168.3	2.692	1.59	247.9	11.6	506.1	43.5	12.68	0.54	4.38	0.72	9.21	2.09	0.99
3	164.5	1.993	1.49	302.6	9.5	564.32	48.5	12.85	0.3	2.78	0.94	7.29	2.64	0.72
4	164.3	1.801	1.48	278.72	9.5	614.95	50	14.8	0.26	2.61	0.95	9.87	2.38	0.68
5	163.2	2	1.59	280.4	9.4	557.25	47.28	14.76	0.31	2.84	0.95	8.47	2.43	0.83
6	151.3	1.935	1.49	275.14	9.4	628.56	52.4	14.76	0.5	3.85	0.96	8.48	2.43	0.54
7	161.2	2.73	1.55	256.3	9.5	539.3	46.2	15.84	0.21	2.12	0.94	8.74	2.36	0.84
8	158.9	2.179	1.38	292.12	8.3	543.87	49.6	14.75	0.23	2.34	1.21	7.2	2.35	0.85
9	158.9	1.391	1.42	276.2	8.6	593.22	53.4	14.75	0.14	4.85	1.16	7.63	3.12	0.52
10	162.9	2.12	1.51	268.31	11.3	632.25	50.6	14.74	0.24	2.38	0.74	8.67	3.13	0.74
11	154.8	2.179	1.41	265.46	8.1	650.86	52.6	15.81	0.61	2.79	1.25	7.34	3.17	0.97
12	154.7	2.198	1.64	324.28	11.6	538.24	45.8	15.43	0.28	2.69	0.74	9.28	2.67	0.78
13	154.6	2.17	1.4	315.47	9.5	612.38	49.2	14.95	0.33	2.97	0.94	9.32	2.67	0.77

14	154.3	2.05	1.43	297.48	8.2	648.92	49.8	14.37	0.43	3.46	1.24	9.34	2.96	0.74
15	153.4	1.801	1.52	312.54	10	672.62	48.2	15.64	0.24	2.43	0.87	7.31	3.24	0.72
16	152.3	1.57	1.42	304.53	7.6	654.32	52.7	14.24	0.26	2.58	1.29	9.53	3.49	0.83
17	158.7	2.583	1.59	247.32	11.2	513.72	46.9	15.08	0.25	2.46	0.81	7.06	3.34	0.69
18	149.2	2.038	1.49	276.8	10.3	514.81	47.6	15.05	0.4	3.38	0.85	9.72	3.42	0.69
19	148.7	2.37	1.49	273.7	9.6	537.68	48.9	14.85	0.37	3.18	0.94	8.29	3.43	0.68
20	148.5	1.28	1.52	329.64	11.8	628.48	54.2	13.62	0.36	3.13	1.3	9.22	4.13	0.82
21	147.5	2.038	1.37	268	9.8	638.64	50.8	13.67	0.32	2.84	0.9	7.1	3.26	0.72
22	147.2	2.403	1.37	337.68	10.6	533.32	47.8	14.25	0.21	2.12	0.83	7.63	2.12	0.72
23	145.6	1.62	1.31	359.78	8.3	647.6	55.2	15.62	0.57	4.57	1.23	8.37	4.83	0.79
24	145.2	2.435	1.39	322.8	10.8	568.34	48.6	14.76	0.34	3.12	0.81	9.27	2.67	0.84
25	143.3	1.801	1.48	356.42	9.1	590.76	47.6	13.56	0.48	3.84	1.12	7.18	2.37	0.84
26	142.5	1.621	1.32	358.9	9.4	564.32	52.8	14.27	0.52	4.23	0.98	8.42	2.36	0.84
27	142.3	1.93	1.57	375.42	10.5	616.03	53.1	14.84	0.28	2.71	0.83	7.41	2.45	0.81
28	142.3	2.15	1.62	265.32	10.5	559.44	45.2	14.84	0.29	2.72	0.84	7.47	2.56	0.79
29	141.6	2.237	1.42	305.9	9.4	528.92	48.2	14.49	0.23	2.35	0.97	9.62	2.13	0.97
30	138.4	1.929	1.39	321.6	6.5	626.92	52.6	14.46	0.28	2.65	1.39	9.63	2.15	0.95
31	137.2	1.46	1.39	395.6	7.7	657.89	54.8	13.36	0.47	3.62	1.26	9.38	2.29	0.87
32	136.9	1.891	1.21	304.15	7.7	639.03	52.8	13.29	0.51	3.95	1.26	10.71	2.31	0.87
33	136.8	2.038	1.42	259.96	8.5	537.25	49	13.68	0.53	4.28	1.16	8.12	2.74	0.75
34	136.4	2.19	1.57	254.6	8.9	543.89	49.6	13.57	0.53	4.32	1.13	10.72	2.37	0.84
35	136.1	2.237	1.34	354.74	8.9	657.94	50.2	13.56	0.43	3.47	1.14	10.73	2.37	0.75
36	135	1.243	1.43	298.7	7.6	703.73	56.2	13.28	0.58	4.73	1.28	10.28	4.73	0.74
37	134.7	1.76	1.27	385.8	8.2	633.45	52.8	15.27	0.36	3.16	1.24	8.12	2.85	0.75
38	134.5	1.78	1.38	324.56	7.5	543.2	54.1	14.74	0.37	3.16	1.3	10.75	3.29	0.69
39	134.5	1.87	1.35	348.67	8.5	654.13	52.4	14.69	0.34	2.98	1.19	10.81	2.96	0.74
40	134.2	1.737	1.52	267.15	8.8	707.89	52.1	13.52	0.51	3.92	1.15	8.19	2.64	0.79
41	134.2	1.467	1.34	353.95	7.3	719.29	54.9	13.49	0.57	4.64	1.35	8.25	3.12	0.52
42	134.2	2.03	1.35	347.85	9.6	538.98	47.5	15.37	0.54	4.32	1.35	8.11	3.57	0.66

43	134.1	1.544	1.37	332.32	7.4	687.68	54.7	12.79	0.59	4.83	1.34	9.74	4.12	0.57
44	172.5	1.97	1.53	340.36	8.8	682.43	53.4	14.65	0.34	3.08	1.15	8.18	3.14	0.73
45	133.1	1.467	1.37	334.8	8.8	648.96	50.9	14.63	0.53	4.32	1.16	10.52	3.53	0.67
46	132.6	1.243	1.27	376.8	7.9	728.27	52.8	13.27	0.59	4.78	1.26	10.54	4.25	0.54
47	132.5	1.24	1.28	376.5	7.9	602.97	53.7	13.24	0.59	4.8	1.26	8.66	4.32	0.53
48	132.4	1.621	1.54	257.87	7.6	534.4	49.8	14.98	0.25	2.46	1.26	8.26	2.18	0.89
49	129.8	1.21	1.28	376.8	7.2	687.98	54.8	14.54	0.64	4.96	1.38	8.14	2.67	0.75
50	128.6	1.577	1.26	387.4	7.6	581.21	53.1	15.24	0.54	4.4	1.27	8.92	3.16	0.72
51	128.6	1.76	1.26	387.6	7.6	585.56	52.9	15.04	0.64	4.87	1.27	8.93	3.21	0.72
52	126.9	1.256	1.28	376.8	7.3	734.67	56.4	13.21	0.73	5.48	1.36	8.14	3.63	0.65
53	126.5	1.692	1.24	392.9	8.2	587.69	51.8	14.37	0.67	5.12	1.24	10.11	2.98	0.74
54	126.5	1.75	1.24	393.69	8.2	632.12	52.5	14.36	0.31	2.84	1.25	10.11	2.98	0.74
55	126.4	1.54	1.51	269.5	8.9	615.7	52.6	12.43	0.64	4.91	1.15	8.56	3.12	0.74
56	126.3	1.487	1.28	367.24	9.9	684.25	52.8	12.38	0.64	4.92	0.87	8.16	2.27	0.74
57	124.9	1.23	1.57	254.6	7.2	649.76	52.8	14.84	0.68	5.25	1.38	8.98	3.82	0.64
58	124.8	1.339	1.27	380.98	7.2	684.57	55.5	14.56	0.71	5.27	1.38	9.15	3.85	0.63
59	124.5	1.27	1.44	354.87	9.2	593.18	54.8	14.36	0.71	5.29	1.06	9.16	3.95	0.59
60	124.3	1.628	1.26	387.9	5.9	721.23	56.4	14.36	0.72	5.39	1.5	9.16	3.87	0.62
61	123.5	1.43	1.25	388.6	5.8	756.8	53.4	12.65	0.72	5.42	1.54	9.18	3.94	0.62
62	123.4	1.342	1.25	389.5	5.6	673.25	55.1	12.56	0.72	5.43	1.54	9.21	3.85	0.62
63	123.4	1.54	1.47	286.2	8.5	612.77	55.2	14.28	0.6	4.83	1.18	8.26	2.95	0.74
64	123.3	1.94	1.29	364.2	8.2	594.27	49.5	13.48	0.35	3.12	1.39	9.26	3.48	0.68
65	121.3	1.265	1.19	397.9	6.3	704.56	55.2	12.87	0.67	5.18	1.43	9.37	4.03	0.58
66	120.6	1.27	1.18	398.4	6.2	768.34	55.6	12.85	0.67	5.2	1.45	9.37	4.05	0.57
67	120.5	1.32	1.27	398.6	5.9	674.35	55.6	12.85	0.72	5.45	1.47	8.27	4.12	0.57
68	120	1.54	1.33	248.31	8.2	647.6	53.5	13.54	0.53	4.28	1.24	9.4	4.13	0.54
69	119.2	1.801	1.37	247.15	6.9	614.95	45.8	15.63	0.41	3.43	1.38	9.12	3.21	0.98
70	116.2	1.37	1.28	376.43	5.2	699.76	54.7	13.46	0.47	3.83	1.83	8.29	3.52	0.67
71	115.7	1.432	1.41	310.88	6.7	683.45	54.6	12.87	0.48	3.84	1.38	8.93	3.54	0.67

72	115.6	1.467	1.27	376.9	5.6	759.98	58.5	14.83	0.76	5.67	1.57	8.93	3.65	0.64
73	115.4	1.324	1.33	385.48	5.4	698.35	54.2	14.95	0.76	5.71	1.58	8.94	3.67	0.64
74	115.3	1.57	0.6	410.2	6.4	635.63	50.9	13.75	0.67	5.13	1.43	9.98	3.59	0.65
75	114.6	1.577	1.45	214.4	5.9	695.32	52.1	13.74	0.75	5.64	1.5	10.63	4.37	0.52
76	113.5	1.75	1.36	342.12	6.6	714.28	52.1	13.57	0.38	3.24	1.39	8.32	4.33	0.53
77	113.2	1.23	1.17	296.5	5.3	788.9	52.7	13.74	0.81	5.82	1.61	7.47	4.37	0.87

Table D. Biochemical parameters of seropositive RA patients

	ROS (% of control)	MDA (nmol /ml)	Carbonyl (nmoles/mg protein)	T-SH (µM)	Tail length (µm)	FRAP (µM)	% quenching of DPPH	NO (µM)	VITC (mg/dl)	GSH (nmoles/mg Hb)	SOD (U/mg Hb)	Catalase (U/mg Hb)	GR (U/g Hb)	GST (U/mg Hb)
1	168.4	2.76	1.59	248.6	11.6	502.84	45.8	15.57	0.53	4.28	0.72	7.34	2.35	0.85
2	164.8	2.621	1.53	262.64	11.3	526.79	45.8	15.04	0.57	4.56	0.78	7.94	2.15	0.95
3	162.3	2.224	1.48	278.75	10.3	594.58	51	14.83	0.33	2.97	0.84	7.83	2.43	0.81
4	159.6	2.32	1.47	286.75	8.4	568.14	46.7	14.83	0.29	2.73	1.2	7.24	0.54	0.76
5	158.9	2.692	1.5	270.68	12.6	504.54	48.6	15.86	0.24	2.37	0.67	9.64	2.29	0.87
6	154.9	2.57	1.41	312.5	8.5	553.99	48.2	14.85	0.21	2.17	1.18	9.28	2.6	0.79
7	153.1	1.987	1.58	254.15	9.6	510.46	46.2	15.28	0.47	3.68	0.93	8.29	3.06	0.74
8	152.3	2.57	1.48	278.49	9.5	535.08	50.5	14.87	0.34	3.04	0.94	10.92	3.28	0.69
9	148.7	2.57	1.5	269.8	9.6	588.82	49.2	14.85	0.39	3.28	0.94	8.28	3.43	0.69
10	148.6	1.935	1.46	286.76	8.5	518.08	47.3	14.37	0.41	3.43	1.18	8.29	3.46	0.68
11	147.8	1.98	1.46	287.14	7.5	612.87	49.8	14.38	0.39	3.38	1.32	7.17	3.47	0.68
12	147.5	2.179	1.64	234.1	8.9	581.21	48.4	14.38	0.29	2.74	1.14	7.37	4.03	0.58
13	146.9	2.435	1.47	284.63	10.6	561.61	46.8	14.21	0.21	2.15	0.83	7.63	2.12	0.97
14	146.9	2.18	1.36	343.34	10.5	631.02	50.2	13.75	0.34	3.08	0.83	7.38	3.65	0.64
15	146.8	1.325	1.37	327.9	9.2	582.39	52.9	13.63	0.34	3.12	1.105	7.39	2.76	0.75
16	143.8	2.391	1.63	246.24	9.3	551.82	47.2	13.84	0.45	3.54	1.03	10.82	2.66	0.78

17	143.5	2.403	1.38	324.4	9.6	568.37	46.9	13.84	0.46	3.57	0.91	7.05	2.67	0.78
18	143.5	2.378	1.28	367.8	9.6	535.49	48.7	13.82	0.47	3.62	0.93	11.28	2.67	0.78
19	142.8	2.378	1.37	334.2	10.7	563.58	48.6	13.28	0.38	3.27	0.82	7.53	4.16	0.54
20	142.6	2.06	1.28	365.64	10.6	607.98	49.5	13.28	0.32	2.94	0.82	7.54	2.67	1.06
21	142.5	2.05	1.37	332.32	8.3	568.14	48.5	15.7	0.26	2.64	1.22	7.64	3.76	0.64
22	142.5	2.13	1.38	324.53	8.3	574.37	49.8	14.3	0.27	2.64	1.224	7.68	3.78	0.64
23	142.3	2.134	1.54	262.64	9.2	555.08	50.5	15.03	0.41	3.42	1.08	7.93	2.19	0.53
24	137.5	1.5	1.45	292.12	8.6	635.63	53.5	13.74	0.54	4.52	1.16	7.94	2.15	0.94
25	136.5	1.891	1.36	343.04	9.2	573.65	49.6	13.73	0.43	3.48	1.1	7.98	2.19	0.87
26	134.8	2.012	1.58	375.46	10.2	696.58	52.7	14.58	0.52	4.28	0.85	10.14	2.75	0.75
27	132.4	1.935	1.32	357.64	7.6	698.76	52.9	15.02	0.25	2.46	1.26	8.26	2.16	0.92
28	132	1.95	1.28	249.24	9.3	521.92	45.6	14.85	0.55	4.55	1.02	8.37	3.46	0.68
29	131.2	1.942	1.28	368.74	9.3	685.95	52.4	14.85	0.58	4.73	1.02	8.37	3.46	0.68
30	129.8	1.935	1.32	358.6	8.4	785.45	50.3	15.31	0.3	2.76	1.2	8.38	3.47	0.68
31	129.8	1.692	1.37	328.3	7.6	625.69	50.8	15.27	0.54	4.37	1.27	8.76	3.15	0.73
32	128.3	1.628	1.37	332.32	7.4	594.38	53.4	14.35	0.57	4.58	1.34	10.15	3.27	0.7
33	127.8	1.84	1.28	254.7	7.3	584.47	44.2	14.33	0.67	5.14	0.93	10.26	3.28	0.7
34	125.9	1.57	1.41	315.2	7.6	637.25	50.7	14.25	0.38	3.27	1.28	9.42	3.25	0.72
35	125.6	1.61	1.39	321	7.6	647.6	54.2	14.25	0.61	4.86	1.29	9.42	3.26	0.72
36	125.6	1.737	1.39	324.28	7.5	713.43	51.6	12.48	0.65	5.12	1.3	9.45	2.95	0.74
37	124.9	1.85	1.14	398.8	7.5	589.45	50.1	14.62	0.45	3.51	1.31	9.48	2.94	0.88
38	119.5	1.83	1.48	284.08	9.2	612.37	56.5	14.27	0.66	5.12	1.11	8.32	3.49	0.68
39	116.8	1.358	1.28	368.7	5.3	797.97	56.7	13.46	0.42	3.45	1.64	8.28	3.49	0.67
40	115.3	1.76	1.23	395.47	5.9	685.67	53.8	13.46	0.78	5.73	1.49	10.38	3.68	0.64
41	113.6	1.76	1.27	386.5	6.6	638.33	46.9	13.58	0.52	4.16	1.24	8.29	3.31	0.69
42	113.5	1.64	1.46	290.5	6.8	615.75	48.9	12.76	0.64	4.87	1.38	8.45	3.64	0.65
43	113.2	1.62	1.37	340.36	6.5	643.28	52.6	13.23	0.74	5.62	1.41	9.03	4.36	0.74

Table E. Biochemical parameters of RA patients with DAS<2.4

	ROS (% of control)	MDA (nmol /ml)	Carbonyl (nmoles/mg protein)	T-SH (µM)	Tail length (µm)	FRAP (µM)	% quenching of DPPH	NO (µM)	VITC (mg/dl)	GSH (nmoles/mg Hb)	SOD (U/mg Hb)	Catalase (U/mg Hb)	GR (U/g Hb)	GST (U/mg Hb)
1	133.6	2.54	1.54	324.17	7.6	515.9	45.6	12.85	0.31	2.04	0.7	9.14	2.13	0.5
2	164.5	1.993	1.49	302.6	9.5	564.32	48.5	12.85	0.3	2.78	0.94	7.29	2.64	0.72
3	164.3	1.801	1.48	278.72	9.5	614.95	50	14.8	0.26	2.61	0.95	9.87	2.38	0.68
4	163.2	2	1.59	280.4	9.4	557.25	47.28	14.76	0.31	2.84	0.95	8.47	2.43	0.83
5	151.3	1.935	1.49	275.14	9.4	628.56	52.4	14.76	0.5	3.85	0.96	8.48	2.43	0.54
6	162.3	2.224	1.48	278.75	10.3	594.58	51	14.83	0.33	2.97	0.84	7.83	2.43	0.81
7	158.9	1.391	1.42	276.2	8.6	593.22	53.4	14.75	0.14	4.85	1.16	7.63	3.12	0.52
8	154.8	2.179	1.41	265.46	8.1	650.86	52.6	15.81	0.61	2.79	1.25	7.34	3.17	0.97
9	153.4	1.801	1.52	312.54	10	672.62	48.2	15.64	0.24	2.43	0.87	7.31	3.24	0.72
10	152.3	1.57	1.42	304.53	7.6	654.32	52.7	14.24	0.26	2.58	1.29	9.53	3.49	0.83
11	148.5	1.28	1.37	329.64	11.8	628.48	54.2	13.62	0.36	3.13	1.3	9.22	4.13	0.82
12	147.8	1.98	1.46	287.14	7.5	612.87	49.8	14.38	0.39	3.38	1.32	7.17	3.47	0.68
13	146.9	2.18	1.36	343.34	10.5	631.02	50.2	13.75	0.34	3.08	0.83	7.38	3.65	0.64
14	146.8	1.325	1.37	327.9	9.2	582.39	52.9	13.63	0.34	3.12	1.105	7.39	2.76	0.75
15	145.6	1.62	1.31	359.78	8.3	647.6	55.2	15.62	0.57	4.57	1.23	8.37	4.83	0.79
16	143.3	1.801	1.48	356.42	9.1	590.76	47.6	13.56	0.48	3.84	1.12	7.18	2.37	0.84
17	142.5	1.621	1.32	358.9	9.4	564.32	52.8	14.27	0.52	4.23	0.98	8.42	2.36	0.84
18	137.5	1.5	1.45	292.12	8.6	635.63	53.5	13.74	0.54	4.52	1.16	7.94	2.15	0.94
19	137.2	1.46	1.39	395.6	7.7	657.89	54.8	13.36	0.47	3.62	1.26	9.38	2.29	0.87
20	135	1.243	1.43	298.7	7.6	703.73	56.2	13.28	0.58	4.73	1.28	10.28	4.73	0.74
21	134.2	1.737	1.52	267.15	8.8	707.89	52.1	13.52	0.51	3.92	1.15	8.19	2.64	0.79
22	134.2	1.467	1.34	353.95	7.3	719.29	54.9	13.49	0.57	4.64	1.35	8.25	3.12	0.52
23	134.1	1.544	1.37	332.32	7.4	687.68	54.7	12.79	0.59	4.83	1.34	9.74	4.12	0.57

24	133.1	1.467	1.37	334.8	8.8	648.96	50.9	14.63	0.53	4.32	1.16	10.52	3.53	0.67
25	132.6	1.243	1.27	376.8	7.9	728.27	52.8	13.27	0.59	4.78	1.26	10.54	4.25	0.54
26	132.5	1.24	1.28	376.5	7.9	602.97	53.7	13.24	0.59	4.8	1.26	8.66	4.32	0.53
27	129.8	1.21	1.28	376.8	7.2	687.98	54.8	14.54	0.64	4.96	1.38	8.14	2.67	0.75
28	126.9	1.256	1.28	376.8	7.3	734.67	56.4	13.21	0.73	5.48	1.36	8.14	3.63	0.65
29	126.4	1.54	1.51	269.5	8.9	615.7	52.6	12.43	0.64	4.91	1.15	8.56	3.12	0.74
30	126.3	1.487	1.28	367.24	9.9	684.25	52.8	12.38	0.64	4.92	0.87	8.16	2.27	0.74
31	124.9	1.23	1.57	254.6	7.2	649.76	52.8	14.84	0.68	5.25	1.38	8.98	3.82	0.64
32	124.8	1.339	1.27	380.98	7.2	684.57	55.5	14.56	0.71	5.27	1.38	9.15	3.85	0.63
33	124.5	1.27	1.44	354.87	9.2	593.18	54.8	14.36	0.71	5.29	1.06	9.16	3.95	0.59
34	124.3	1.628	1.26	387.9	5.9	721.23	56.4	14.36	0.72	5.39	1.5	9.16	3.87	0.62
35	123.5	1.43	1.25	388.6	5.8	756.8	53.4	12.65	0.72	5.42	1.54	9.18	3.94	0.62
36	123.4	1.342	1.25	389.5	5.6	673.25	55.1	12.56	0.72	5.43	1.54	9.21	3.85	0.62
37	123.4	1.54	1.47	286.2	8.5	612.77	55.2	14.28	0.6	4.83	1.18	8.26	2.95	0.74
38	121.3	1.265	1.19	397.9	6.3	704.56	55.2	12.87	0.67	5.18	1.43	9.37	4.03	0.58
39	120.6	1.27	1.18	398.4	6.2	768.34	55.6	12.85	0.67	5.2	1.45	9.37	4.05	0.57
40	120.5	1.32	1.27	398.6	5.9	674.35	55.6	12.85	0.72	5.45	1.47	8.27	4.12	0.57
41	120	1.54	1.33	248.31	8.2	647.6	53.5	13.54	0.53	4.28	1.24	9.4	4.13	0.54
42	119.5	1.83	1.48	284.08	9.2	612.37	56.5	14.27	0.66	5.12	1.11	8.32	3.49	0.68
43	116.8	1.358	1.28	368.7	5.3	797.97	56.7	13.46	0.42	3.45	1.64	8.28	3.49	0.67
44	116.2	1.37	1.28	376.43	5.2	699.76	54.7	13.46	0.47	3.83	1.83	8.29	3.52	0.67
45	115.6	1.467	1.27	376.9	5.6	759.98	58.5	14.83	0.76	5.67	1.57	8.93	3.65	0.64
46	115.4	1.324	1.33	385.48	5.4	698.35	54.2	14.95	0.76	5.71	1.58	8.94	3.67	0.64
47	115.3	1.76	1.23	395.47	5.9	685.67	53.8	13.46	0.78	5.73	1.49	10.38	3.68	0.64
48	115.3	1.57	0.6	410.2	6.4	635.63	50.9	13.75	0.67	5.13	1.43	9.98	3.59	0.65
49	113.2	1.23	1.17	296.5	5.3	788.9	52.7	13.74	0.81	5.82	1.61	7.47	4.37	0.87

Table F. Biochemical parameters of RA patients with DAS>2.4

	ROS (% of control)	MDA (nmol /ml)	Carbonyl (nmoles/mg protein)	T-SH (µM)	Tail length (µm)	FRAP (µM)	% quenching of DPPH	NO (µM)	VITC (mg/dl)	GSH (nmoles/mg Hb)	SOD (U/mg Hb)	Catalase (U/mg Hb)	GR (U/g Hb)	GST (U/mg Hb)
1	168.4	2.76	1.59	248.6	11.6	502.84	45.8	15.57	0.53	4.28	0.72	7.34	2.35	0.85
2	168.3	2.692	1.59	247.9	11.6	506.1	43.5	12.68	0.54	4.38	0.72	9.21	2.09	0.99
3	164.8	2.621	1.53	262.64	11.3	526.79	45.8	15.04	0.57	4.56	0.78	7.94	2.15	0.95
4	161.2	2.73	1.55	256.3	9.5	539.3	46.2	15.84	0.21	2.12	0.94	8.74	2.36	0.84
5	159.6	2.32	1.47	286.75	8.4	568.14	46.7	14.83	0.29	2.73	1.2	7.24	0.54	0.76
6	158.9	2.179	1.38	292.12	8.3	543.87	49.6	14.75	0.23	2.34	1.21	7.2	2.35	0.85
7	158.9	2.692	1.5	270.68	12.6	504.54	48.6	15.86	0.24	2.37	0.67	9.64	2.29	0.87
8	162.9	2.12	1.51	268.31	11.3	632.25	50.6	14.74	0.24	2.38	0.74	8.67	3.13	0.74
9	154.9	2.57	1.41	312.5	8.5	553.99	48.2	14.85	0.21	2.17	1.18	9.28	2.6	0.79
10	154.7	2.198	1.64	324.28	11.6	538.24	45.8	15.43	0.28	2.69	0.74	9.28	2.67	0.78
11	154.6	2.17	1.4	315.47	9.5	612.38	49.2	14.95	0.33	2.97	0.94	9.32	2.67	0.77
12	154.3	2.05	1.43	297.48	8.2	648.92	49.8	14.37	0.43	3.46	1.24	9.34	2.96	0.74
13	153.1	1.987	1.58	254.15	9.6	510.46	46.2	15.28	0.47	3.68	0.93	8.29	3.06	0.74
14	152.3	2.57	1.48	278.49	9.5	535.08	50.5	14.87	0.34	3.04	0.94	10.92	3.28	0.69
15	158.7	2.583	1.59	247.32	11.2	513.72	46.9	15.08	0.25	2.46	0.81	7.06	3.34	0.69
16	149.2	2.038	1.49	276.8	10.3	514.81	47.6	15.05	0.4	3.38	0.85	9.72	3.42	0.69
17	148.7	2.57	1.5	269.8	9.6	588.82	49.2	14.85	0.39	3.28	0.94	8.28	3.43	0.69
18	148.7	2.37	1.49	273.7	9.6	537.68	48.9	14.85	0.37	3.18	0.94	8.29	3.43	0.68
19	148.6	1.935	1.46	286.76	8.5	518.08	47.3	14.37	0.41	3.43	1.18	8.29	3.46	0.68
20	147.5	2.038	1.37	268	9.8	638.64	50.8	13.67	0.32	2.84	0.9	7.1	3.26	0.72
21	147.5	2.179	1.64	234.1	8.9	581.21	48.4	14.38	0.29	2.74	1.14	7.37	4.03	0.58
22	147.2	2.403	1.52	337.68	10.6	533.32	47.8	14.25	0.21	2.12	0.83	7.63	2.12	0.72
23	146.9	2.435	1.47	284.63	10.6	561.61	46.8	14.21	0.21	2.15	0.83	7.63	2.12	0.97
24	145.2	2.435	1.39	322.8	10.8	568.34	48.6	14.76	0.34	3.12	0.81	9.27	2.67	0.84
25	143.8	2.391	1.63	246.24	9.3	551.82	47.2	13.84	0.45	3.54	1.03	10.82	2.66	0.78

26	143.5	2.403	1.38	324.4	9.6	568.37	46.9	13.84	0.46	3.57	0.91	7.05	2.67	0.78
27	143.5	2.378	1.28	367.8	9.6	535.49	48.7	13.82	0.47	3.62	0.93	11.28	2.67	0.78
28	142.8	2.378	1.37	334.2	10.7	563.58	48.6	13.28	0.38	3.27	0.82	7.53	4.16	0.54
29	142.6	2.06	1.28	365.64	10.6	607.98	49.5	13.28	0.32	2.94	0.82	7.54	2.67	1.06
30	142.5	2.05	1.37	332.32	8.3	568.14	48.5	15.7	0.26	2.64	1.22	7.64	3.76	0.64
31	142.5	2.13	1.38	324.53	8.3	574.37	49.8	14.3	0.27	2.64	1.224	7.68	3.78	0.64
32	142.3	1.93	1.57	375.42	10.5	616.03	53.1	14.84	0.28	2.71	0.83	7.41	2.45	0.81
33	142.3	2.15	1.62	265.32	10.5	559.44	45.2	14.84	0.29	2.72	0.84	7.47	2.56	0.79
34	142.3	2.134	1.54	262.64	9.2	555.08	50.5	15.03	0.41	3.42	1.08	7.93	2.19	0.53
35	141.6	2.237	1.42	305.9	9.4	528.92	48.2	14.49	0.23	2.35	0.97	9.62	2.13	0.97
36	138.4	1.929	1.39	321.6	6.5	626.92	52.6	14.46	0.28	2.65	1.39	9.63	2.15	0.95
37	136.9	1.891	1.21	304.15	7.7	639.03	52.8	13.29	0.51	3.95	1.26	10.71	2.31	0.87
38	136.8	2.038	1.42	259.96	8.5	537.25	49	13.68	0.53	4.28	1.16	8.12	2.74	0.75
39	136.5	1.891	1.36	343.04	9.2	573.65	49.6	13.73	0.43	3.48	1.1	7.98	2.19	0.87
40	136.4	2.19	1.57	254.6	8.9	543.89	49.6	13.57	0.53	4.32	1.13	10.72	2.37	0.84
41	136.1	2.237	1.34	354.74	8.9	657.94	50.2	13.56	0.43	3.47	1.14	10.73	2.37	0.75
42	134.8	2.012	1.58	375.46	10.2	696.58	52.7	14.58	0.52	4.28	0.85	10.14	2.75	0.75
43	134.7	1.76	1.27	385.8	8.2	633.45	52.8	15.27	0.36	3.16	1.24	8.12	2.85	0.75
44	134.5	1.78	1.38	324.56	7.5	543.2	54.1	14.74	0.37	3.16	1.3	10.75	3.29	0.69
45	134.5	1.87	1.35	348.67	8.5	654.13	52.4	14.69	0.34	2.98	1.19	10.81	2.96	0.74
46	134.2	2.03	1.35	347.85	9.6	538.98	47.5	15.37	0.54	4.32	1.35	8.11	3.57	0.66
47	172.5	1.97	1.53	340.36	8.8	682.43	53.4	14.65	0.34	3.08	1.15	8.18	3.14	0.73
48	132.4	1.935	1.32	357.64	7.6	698.76	52.9	15.02	0.25	2.46	1.26	8.26	2.16	0.92
49	132.4	1.621	1.54	257.87	7.6	534.4	49.8	14.98	0.25	2.46	1.26	8.26	2.18	0.89
50	132	1.95	1.28	249.24	9.3	521.92	45.6	14.85	0.55	4.55	1.02	8.37	3.46	0.68
51	131.2	1.942	1.28	368.74	9.3	685.95	52.4	14.85	0.58	4.73	1.02	8.37	3.46	0.68
52	129.8	1.935	1.32	358.6	8.4	785.45	50.3	15.31	0.3	2.76	1.2	8.38	3.47	0.68
53	129.8	1.692	1.37	328.3	7.6	625.69	50.8	15.27	0.54	4.37	1.27	8.76	3.15	0.73
54	128.6	1.577	1.26	387.4	7.6	581.21	53.1	15.24	0.54	4.4	1.27	8.92	3.16	0.72

55	128.6	1.76	1.26	387.6	7.6	585.56	52.9	15.04	0.64	4.87	1.27	8.93	3.21	0.72
56	128.3	1.628	1.37	332.32	7.4	594.38	53.4	14.35	0.57	4.58	1.34	10.15	3.27	0.7
57	127.8	1.84	1.28	254.7	7.3	584.47	44.2	14.33	0.67	5.14	0.93	10.26	3.28	0.7
58	126.5	1.692	1.24	392.9	8.2	587.69	51.8	14.37	0.67	5.12	1.24	10.11	2.98	0.74
59	126.5	1.75	1.24	393.69	8.2	632.12	52.5	14.36	0.31	2.84	1.25	10.11	2.98	0.74
60	125.9	1.57	1.41	315.2	7.6	637.25	50.7	14.25	0.38	3.27	1.28	9.42	3.25	0.72
61	125.6	1.61	1.39	321	7.6	647.6	54.2	14.25	0.61	4.86	1.29	9.42	3.26	0.72
62	125.6	1.737	1.39	324.28	7.5	713.43	51.6	12.48	0.65	5.12	1.3	9.45	2.95	0.74
63	124.9	1.85	1.14	398.8	7.5	589.45	50.1	14.62	0.45	3.51	1.31	9.48	2.94	0.88
64	123.3	1.94	1.29	364.2	8.2	594.27	49.5	13.48	0.35	3.12	1.39	9.26	3.48	0.68
65	119.2	1.801	1.37	247.15	6.9	614.95	45.8	15.63	0.41	3.43	1.38	9.12	3.21	0.98
66	115.7	1.432	1.41	310.88	6.7	683.45	54.6	12.87	0.48	3.84	1.38	8.93	3.54	0.67
67	114.6	1.577	1.45	214.4	5.9	695.32	52.1	13.74	0.75	5.64	1.5	10.63	4.37	0.52
68	113.6	1.76	1.27	386.5	6.6	638.33	46.9	13.58	0.52	4.16	1.24	8.29	3.31	0.69
69	113.5	1.75	1.36	342.12	6.6	714.28	52.1	13.57	0.38	3.24	1.39	8.32	4.33	0.53
70	113.5	1.64	1.46	290.5	6.8	615.75	48.9	12.76	0.64	4.87	1.38	8.45	3.64	0.65
71	113.2	1.62	1.37	340.36	6.5	643.28	52.6	13.23	0.74	5.62	1.41	9.03	4.36	0.74

Table G. Biochemical parameters of newly diagnosed (ND) RA patients

	**ROS (% of control)**	**MDA (nmol/ml)**	**Carbonyl (nmoles/mg protein)**	**T-SH (μM)**	**Tail length (μm)**	**FRAP (μM)**	**% quenching of DPPH**	**NO (μM)**	**VITC (mg/dl)**	**GSH (nmoles/mg Hb)**	**SOD (U/mg Hb)**	**Catalase (U/mg Hb)**	**GR (U/g Hb)**	**GST (U/mg Hb)**
1	133.6	2.54	1.54	324.17	7.6	515.9	45.6	12.85	0.31	2.04	0.7	9.14	2.13	0.5
2	164.5	1.993	1.49	302.6	9.5	564.32	48.5	12.85	0.3	2.78	0.94	7.29	2.64	0.72
3	164.3	1.801	1.48	278.72	9.5	614.95	50	14.8	0.26	2.61	0.95	9.87	2.38	0.68
4	151.3	1.935	1.49	275.14	9.4	628.56	52.4	14.76	0.5	3.85	0.96	8.48	2.43	0.54
5	159.6	2.32	1.47	286.75	8.4	568.14	46.7	14.83	0.29	2.73	1.2	7.24	0.54	0.76

6	158.9	2.179	1.38	292.12	8.3	543.87	49.6	14.75	0.23	2.34	1.21	7.2	2.35	0.85
7	158.9	1.391	1.42	276.2	8.6	593.22	53.4	14.75	0.14	4.85	1.16	7.63	3.12	0.52
8	162.9	2.12	1.51	268.31	11.3	632.25	50.6	14.74	0.24	2.38	0.74	8.67	3.13	0.74
9	154.8	2.179	1.41	265.46	8.1	650.86	52.6	15.81	0.61	2.79	1.25	7.34	3.17	0.97
10	154.3	2.05	1.43	297.48	8.2	648.92	49.8	14.37	0.43	3.46	1.24	9.34	2.96	0.74
11	152.3	1.57	1.42	304.53	7.6	654.32	52.7	14.24	0.26	2.58	1.29	9.53	3.49	0.83
12	148.5	1.28	1.37	329.64	11.8	628.48	54.2	13.62	0.36	3.13	1.3	9.22	4.13	0.82
13	147.5	2.038	1.37	268	9.8	638.64	50.8	13.67	0.32	2.84	0.9	7.1	3.26	0.72
14	145.6	1.62	1.31	359.78	8.3	647.6	55.2	15.62	0.57	4.57	1.23	8.37	4.83	0.79
15	142.6	2.06	1.28	365.64	10.6	607.98	49.5	13.28	0.32	2.94	0.82	7.54	2.67	1.06
16	142.5	2.05	1.37	332.32	8.3	568.14	48.5	15.7	0.26	2.64	1.22	7.64	3.76	0.64
17	142.3	1.93	1.57	375.42	10.5	616.03	53.1	14.84	0.28	2.71	0.83	7.41	2.45	0.81
18	138.4	1.929	1.39	321.6	6.5	626.92	52.6	14.46	0.28	2.65	1.39	9.63	2.15	0.95
19	137.2	1.46	1.39	395.6	7.7	657.89	54.8	13.36	0.47	3.62	1.26	9.38	2.29	0.87
20	136.9	1.891	1.21	304.15	7.7	639.03	52.8	13.29	0.51	3.95	1.26	10.71	2.31	0.87
21	135	1.243	1.43	298.7	7.6	703.73	56.2	13.28	0.58	4.73	1.28	10.28	4.73	0.74
22	134.8	2.012	1.58	375.46	10.2	696.58	52.7	14.58	0.52	4.28	0.85	10.14	2.75	0.75
23	134.5	1.78	1.38	324.56	7.5	543.2	54.1	14.74	0.37	3.16	1.3	10.75	3.29	0.69
24	134.2	1.467	1.34	353.95	7.3	719.29	54.9	13.49	0.57	4.64	1.35	8.25	3.12	0.52
25	134.1	1.544	1.37	332.32	7.4	687.68	54.7	12.79	0.59	4.83	1.34	9.74	4.12	0.57
26	132.6	1.243	1.27	376.8	7.9	728.27	52.8	13.27	0.59	4.78	1.26	10.54	4.25	0.54
27	132.5	1.24	1.28	376.5	7.9	602.97	53.7	13.24	0.59	4.8	1.26	8.66	4.32	0.53
28	132.4	1.935	1.32	357.64	7.6	698.76	52.9	15.02	0.25	2.46	1.26	8.26	2.16	0.92
29	132.4	1.621	1.54	257.87	7.6	534.4	49.8	14.98	0.25	2.46	1.26	8.26	2.18	0.89
30	131.2	1.942	1.28	368.74	9.3	685.95	52.4	14.85	0.58	4.73	1.02	8.37	3.46	0.68
31	129.8	1.21	1.28	376.8	7.2	687.98	54.8	14.54	0.64	4.96	1.38	8.14	2.67	0.75
32	128.3	1.628	1.37	332.32	7.4	594.38	53.4	14.35	0.57	4.58	1.34	10.15	3.27	0.7
33	126.9	1.256	1.28	376.8	7.3	734.67	56.4	13.21	0.73	5.48	1.36	8.14	3.63	0.65
34	125.9	1.57	1.41	315.2	7.6	637.25	50.7	14.25	0.38	3.27	1.28	9.42	3.25	0.72

35	125.6	1.61	1.39	321	7.6	647.6	54.2	14.25	0.61	4.86	1.29	9.42	3.26	0.72
36	125.6	1.737	1.39	324.28	7.5	713.43	51.6	12.48	0.65	5.12	1.3	9.45	2.95	0.74
37	124.9	1.23	1.57	254.6	7.2	649.76	52.8	14.84	0.68	5.25	1.38	8.98	3.82	0.64
38	124.8	1.339	1.27	380.98	7.2	684.57	55.5	14.56	0.71	5.27	1.38	9.15	3.85	0.63
39	124.3	1.628	1.26	387.9	5.9	721.23	56.4	14.36	0.72	5.39	1.5	9.16	3.87	0.62
40	123.5	1.43	1.25	388.6	5.8	756.8	53.4	12.65	0.72	5.42	1.54	9.18	3.94	0.62
41	123.4	1.342	1.25	389.5	5.6	673.25	55.1	12.56	0.72	5.43	1.54	9.21	3.85	0.62
42	121.3	1.265	1.19	397.9	6.3	704.56	55.2	12.87	0.67	5.18	1.43	9.37	4.03	0.58
43	120.6	1.27	1.18	398.4	6.2	768.34	55.6	12.85	0.67	5.2	1.45	9.37	4.05	0.57
44	120.5	1.32	1.27	398.6	5.9	674.35	55.6	12.85	0.72	5.45	1.47	8.27	4.12	0.57
45	119.5	1.83	1.48	284.08	9.2	612.37	56.5	14.27	0.66	5.12	1.11	8.32	3.49	0.68
46	116.8	1.358	1.28	368.7	5.3	797.97	56.7	13.46	0.42	3.45	1.64	8.28	3.49	0.67
47	116.2	1.37	1.28	376.43	5.2	699.76	54.7	13.46	0.47	3.83	1.83	8.29	3.52	0.67
48	115.7	1.432	1.41	310.88	6.7	683.45	54.6	12.87	0.48	3.84	1.38	8.93	3.54	0.67
49	115.6	1.467	1.27	376.9	5.6	759.98	58.5	14.83	0.76	5.67	1.57	8.93	3.65	0.64
50	115.4	1.324	1.33	385.48	5.4	698.35	54.2	14.95	0.76	5.71	1.58	8.94	3.67	0.64
51	115.3	1.76	1.23	395.47	5.9	685.67	53.8	13.46	0.78	5.73	1.49	10.38	3.68	0.64
52	114.6	1.577	1.45	214.4	5.9	695.32	52.1	13.74	0.75	5.64	1.5	10.63	4.37	0.52
53	113.2	1.23	1.17	296.5	5.3	788.9	52.7	13.74	0.81	5.82	1.61	7.47	4.37	0.87

Table H. Biochemical parameters of RA patients with ≤ 2 Years of disease duration

	ROS (% of control)	MDA (nmol /ml)	Carbonyl (nmoles/mg protein)	T-SH (µM)	Tail length (µm)	FRAP (µM)	% quenching of DPPH	NO (µM)	VITC (mg/dl)	GSH (nmoles/mg Hb)	SOD (U/mg Hb)	Catalase (U/mg Hb)	GR (U/g Hb)	GST (U/mg Hb)
1	164.8	2.621	1.53	262.64	11.3	526.79	45.8	15.04	0.57	4.56	0.78	7.94	2.15	0.95
2	163.2	2	1.59	280.4	9.4	557.25	47.28	14.76	0.31	2.84	0.95	8.47	2.43	0.83
3	162.3	2.224	1.48	278.75	10.3	594.58	51	14.83	0.33	2.97	0.84	7.83	2.43	0.81
4	154.9	2.57	1.41	312.5	8.5	553.99	48.2	14.85	0.21	2.17	1.18	9.28	2.6	0.79

5	154.6	2.17	1.4	315.47	9.5	612.38	49.2	14.95	0.33	2.97	0.94	9.32	2.67	0.77
6	153.1	1.987	1.58	254.15	9.6	510.46	46.2	15.28	0.47	3.68	0.93	8.29	3.06	0.74
7	152.3	2.57	1.48	278.49	9.5	535.08	50.5	14.87	0.34	3.04	0.94	10.92	3.28	0.69
8	149.2	2.038	1.49	276.8	10.3	514.81	47.6	15.05	0.4	3.38	0.85	9.72	3.42	0.69
9	148.7	2.57	1.5	269.8	9.6	588.82	49.2	14.85	0.39	3.28	0.94	8.28	3.43	0.69
10	148.7	2.37	1.49	273.7	9.6	537.68	48.9	14.85	0.37	3.18	0.94	8.29	3.43	0.68
11	148.6	1.935	1.46	286.76	8.5	518.08	47.3	14.37	0.41	3.43	1.18	8.29	3.46	0.68
12	147.8	1.98	1.46	287.14	7.5	612.87	49.8	14.38	0.39	3.38	1.32	7.17	3.47	0.68
13	147.5	2.179	1.64	234.1	8.9	581.21	48.4	14.38	0.29	2.74	1.14	7.37	4.03	0.58
14	146.8	1.325	1.37	327.9	9.2	582.39	52.9	13.63	0.34	3.12	1.105	7.39	2.76	0.75
15	143.8	2.391	1.63	246.24	9.3	551.82	47.2	13.84	0.45	3.54	1.03	10.82	2.66	0.78
16	143.3	1.801	1.48	356.42	9.1	590.76	47.6	13.56	0.48	3.84	1.12	7.18	2.37	0.84
17	142.5	1.621	1.32	358.9	9.4	564.32	52.8	14.27	0.52	4.23	0.98	8.42	2.36	0.84
18	142.5	2.13	1.38	324.53	8.3	574.37	49.8	14.3	0.27	2.64	1.224	7.68	3.78	0.64
19	142.3	2.134	1.54	262.64	9.2	555.08	50.5	15.03	0.41	3.42	1.08	7.93	2.19	0.53
20	137.5	1.5	1.45	292.12	8.6	635.63	53.5	13.74	0.54	4.52	1.16	7.94	2.15	0.94
21	136.5	1.891	1.36	343.04	9.2	573.65	49.6	13.73	0.43	3.48	1.1	7.98	2.19	0.87
22	134.7	1.76	1.27	385.8	8.2	633.45	52.8	15.27	0.36	3.16	1.24	8.12	2.85	0.75
23	134.5	1.87	1.35	348.67	8.5	654.13	52.4	14.69	0.34	2.98	1.19	10.81	2.96	0.74
24	134.2	1.737	1.52	267.15	8.8	707.89	52.1	13.52	0.51	3.92	1.15	8.19	2.64	0.79
25	129.8	1.935	1.32	358.6	8.4	785.45	50.3	15.31	0.3	2.76	1.2	8.38	3.47	0.68
26	129.8	1.692	1.37	328.3	7.6	625.69	50.8	15.27	0.54	4.37	1.27	8.76	3.15	0.73
27	128.6	1.577	1.26	387.4	7.6	581.21	53.1	15.24	0.54	4.4	1.27	8.92	3.16	0.72
28	128.6	1.76	1.26	387.6	7.6	585.56	52.9	15.04	0.64	4.87	1.27	8.93	3.21	0.72
29	126.5	1.692	1.24	392.9	8.2	587.69	51.8	14.37	0.67	5.12	1.24	10.11	2.98	0.74
30	126.5	1.75	1.24	393.69	8.2	632.12	52.5	14.36	0.31	2.84	1.25	10.11	2.98	0.74
31	126.4	1.54	1.51	269.5	8.9	615.7	52.6	12.43	0.64	4.91	1.15	8.56	3.12	0.74
32	124.9	1.85	1.14	398.8	7.5	589.45	50.1	14.62	0.45	3.51	1.31	9.48	2.94	0.88
33	123.4	1.54	1.47	286.2	8.5	612.77	55.2	14.28	0.6	4.83	1.18	8.26	2.95	0.74

34	123.3	1.94	1.29	364.2	8.2	594.27	49.5	13.48	0.35	3.12	1.39	9.26	3.48	0.68
35	120	1.54	1.33	248.31	8.2	647.6	53.5	13.54	0.53	4.28	1.24	9.4	4.13	0.54
36	115.3	1.57	0.6	410.2	6.4	635.63	50.9	13.75	0.67	5.13	1.43	9.98	3.59	0.65
37	113.6	1.76	1.27	386.5	6.6	638.33	46.9	13.58	0.52	4.16	1.24	8.29	3.31	0.69
38	113.5	1.75	1.36	342.12	6.6	714.28	52.1	13.57	0.38	3.24	1.39	8.32	4.33	0.53
39	113.2	1.62	1.37	340.36	6.5	643.28	52.6	13.23	0.74	5.62	1.41	9.03	4.36	0.74

Table I. Biochemical parameters of RA patients with 2-5 years of disease duration

	**ROS (% of control)**	**MDA (nmol /ml)**	**Carbonyl (nmoles/mg protein)**	**T-SH (µM)**	**Tail length (µm)**	**FRAP (µM)**	**% quenching of DPPH**	**NO (µM)**	**VITC (mg/dl)**	**GSH (nmoles/mg Hb)**	**SOD (U/mg Hb)**	**Catalase (U/mg Hb)**	**GR (U/g Hb)**	**GST (U/mg Hb)**
1	168.4	2.76	1.59	248.6	11.6	502.84	45.8	15.57	0.53	4.28	0.72	7.34	2.35	0.85
2	168.3	2.692	1.59	247.9	11.6	506.1	43.5	12.68	0.54	4.38	0.72	9.21	2.09	0.99
3	161.2	2.73	1.55	256.3	9.5	539.3	46.2	15.84	0.21	2.12	0.94	8.74	2.36	0.84
4	158.9	2.692	1.5	270.68	12.6	504.54	48.6	15.86	0.24	2.37	0.67	9.64	2.29	0.87
5	154.7	2.198	1.64	324.28	11.6	538.24	45.8	15.43	0.28	2.69	0.74	9.28	2.67	0.78
6	153.4	1.801	1.52	312.54	10	672.62	48.2	15.64	0.24	2.43	0.87	7.31	3.24	0.72
7	158.7	2.583	1.59	247.32	11.2	513.72	46.9	15.08	0.25	2.46	0.81	7.06	3.34	0.69
8	147.2	2.403	1.52	337.68	10.6	533.32	47.8	14.25	0.21	2.12	0.83	7.63	2.12	0.72
9	146.9	2.435	1.47	284.63	10.6	561.61	46.8	14.21	0.21	2.15	0.83	7.63	2.12	0.97
10	146.9	2.18	1.36	343.34	10.5	631.02	50.2	13.75	0.34	3.08	0.83	7.38	3.65	0.64
11	145.2	2.435	1.39	322.8	10.8	568.34	48.6	14.76	0.34	3.12	0.81	9.27	2.67	0.84
12	143.5	2.403	1.38	324.4	9.6	568.37	46.9	13.84	0.46	3.57	0.91	7.05	2.67	0.78
13	143.5	2.378	1.28	367.8	9.6	535.49	48.7	13.82	0.47	3.62	0.93	11.28	2.67	0.78
14	142.8	2.378	1.37	334.2	10.7	563.58	48.6	13.28	0.38	3.27	0.82	7.53	4.16	0.54
15	142.3	2.15	1.62	265.32	10.5	559.44	45.2	14.84	0.29	2.72	0.84	7.47	2.56	0.79
16	141.6	2.237	1.42	305.9	9.4	528.92	48.2	14.49	0.23	2.35	0.97	9.62	2.13	0.97
17	136.8	2.038	1.42	259.96	8.5	537.25	49	13.68	0.53	4.28	1.16	8.12	2.74	0.75

18	136.4	2.19	1.57	254.6	8.9	543.89	49.6	13.57	0.53	4.32	1.13	10.72	2.37	0.84
19	136.1	2.237	1.34	354.74	8.9	657.94	50.2	13.56	0.43	3.47	1.14	10.73	2.37	0.75
20	134.2	2.03	1.35	347.85	9.6	538.98	47.5	15.37	0.54	4.32	1.35	8.11	3.57	0.66
21	172.5	1.97	1.53	340.36	8.8	682.43	53.4	14.65	0.34	3.08	1.15	8.18	3.14	0.73
22	133.1	1.467	1.37	334.8	8.8	648.96	50.9	14.63	0.53	4.32	1.16	10.52	3.53	0.67
23	132	1.95	1.28	249.24	9.3	521.92	45.6	14.85	0.55	4.55	1.02	8.37	3.46	0.68
24	127.8	1.84	1.28	254.7	7.3	584.47	44.2	14.33	0.67	5.14	0.93	10.26	3.28	0.7
25	126.3	1.487	1.28	367.24	9.9	684.25	52.8	12.38	0.64	4.92	0.87	8.16	2.27	0.74
26	124.5	1.27	1.44	354.87	9.2	593.18	54.8	14.36	0.71	5.29	1.06	9.16	3.95	0.59
27	119.2	1.801	1.37	247.15	6.9	614.95	45.8	15.63	0.41	3.43	1.38	9.12	3.21	0.98
28	113.5	1.64	1.46	290.5	6.8	615.75	48.9	12.76	0.64	4.87	1.38	8.45	3.64	0.65
